# β-d-Ribofuranose as a Core with a Phosphodiester Moiety as the Enzyme Recognition Site for Codrug Development

**DOI:** 10.1021/acs.orglett.4c03662

**Published:** 2024-11-14

**Authors:** Jih Ru Hwu, Avijit Panja, Shwu-Chen Tsay, Wen-Chieh Huang, Shu-Yu Lin, Chen-Sheng Yeh, Wu-Chou Su, Li-Xing Yang, Dar-Bin Shieh

**Affiliations:** † Department of Chemistry & Frontier Research Center on Fundamental and Applied Sciences of Matters, National Tsing Hua University, Hsinchu 300, Taiwan; ‡ Institute of Biotechnology and Pharmaceutical Research, National Health Research Institutes, Zhunan, Miaoli County 350401, Taiwan; § Department of Chemistry, National Cheng Kung University, Tainan 701, Taiwan; ∥ Department of Internal Medicine, National Cheng Kung University, Tainan 701, Taiwan; ⊥ Department of Dentistry and Institute of Oral Medicine, National Cheng Kung University, Tainan 701, Taiwan

## Abstract

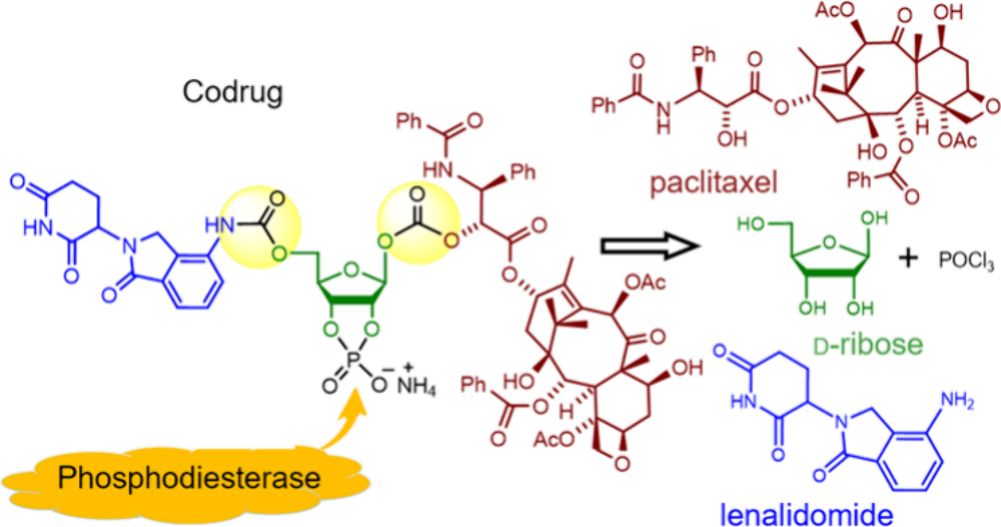

An ideal codrug design should be able to control drug release, offer selectivity during drug delivery, and break down into non-toxic fragments after biodegradation. Our design incorporates d-ribofuranose as the core, with carbamate and carbonate groups as linking joints, a phosphodiester moiety as an enzyme recognition site, and lenalidomide and paclitaxel as the constituent drugs. The codrug synthesis involves seven steps with a 33% overall yield. The target codrug increases its water solubility 685 times versus paclitaxel.

An innovative approach to enhance drug delivery properties is to link drugs together by chemical bonds to form a codrug.^1^ The constituent drugs in a codrug can target the same disease while exerting different therapeutic effects through divergent mechanisms of action.^2a,2b^ Four major factors were taken into consideration in our codrug design: (1) What kind of core moiety, with appropriate geometry and a sufficient number of functional groups, will be used to connect the constituent drugs and the enzyme recognition moiety? (2) What kind of joints are suitable for their installation in the anticancer codrug? (3) How can the constituent drugs be released selectively from the codrug? (4) How can the core residue be non-toxic after the constituent drugs are released?

For the core moiety of codrug **1**, we selected non-toxic β-d-ribofuranose (**3**), as shown in Scheme 1. To the best of our knowledge, β-d-ribofuranose has never been utilized previously in codrug development. This compound is naturally occurring and forms the backbone of RNAs. Its two hydroxyl groups at the C2″ and C3″ positions, which have a *cis* configuration, are ideal for equipment of a phosphodiester moiety as the enzyme recognition site. The remaining hydroxyl groups at the C1″ and C5″ positions are available for attachment to the constituent drugs. For these purposes, we selected a carbamate joint [−NH(C=O)O−] for lenalidomide (**2**, LENA) and a carbonate joint [−O(C=O)O−] for paclitaxel (**4**, PTX) to connect with the ribofuranose core.

**Scheme 1:**
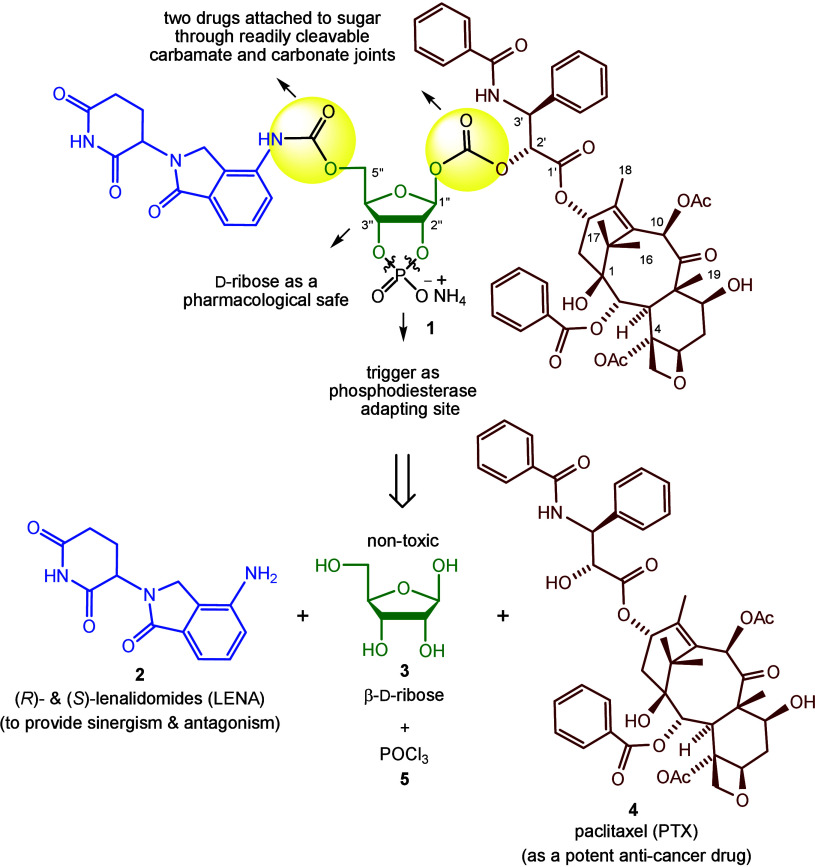
Design of Codrug **1** and Its Retrosynthetic Strategy

Phosphate-containing chemotherapeutic agents often exhibit a preferential interaction with cancer cells.^3−5^ Furthermore, dephosphorylation occurs more readily in cancer cells than in normal cells. This process can be accomplished by phosphodiesterase, which breaks phosphodiester bonds. Therefore, this is recognized with great clinical significance. Consequently, we adopted a phosphodiester group as a crucial component in the codrug to be synthesized.

LENA (**2**) is an immunomodulatory drug with potent antiangiogenic, antitumor, and proapoptotic activities, particularly in multiple myeloma. It is also active against various hematological disorders and is under clinical trials for the treatment for pancreatic carcinoma, chronic lymphocytic leukemia, and lymphoma. LENA contains one stereogenic center, with the *S*-(−) enantiomer being more potent^6,7^ than the *R*-(+) enantiomer. However, the development of single enantiomeric formulations has been discontinued due to rapid racemization *in vivo*.^8^ Therefore, it is marked as a racemic mixture.^9^ Despite its effectiveness against a range of hematologic and solid cancers, its toxicity remains a concern.^10^ Furthermore, a modular phase I study of LENA (**2**) and PTX (**4**) in men with metastatic castration-resistant prostate cancer was conducted. It was used to assess prostate-specific antigen (PSA) kinetics with lead-in compound **2** and the feasibility of their physical combination.^11^

(−)-PTX (**4**) is widely used for the treatment of bladder, breast, esophageal, Kaposi’s sarcoma, lung, melanoma, ovary, and prostate cancers.^12,13^ Its popularity as a chemotherapeutic agent stems from its mechanism of action.^14^ However, it has notable drawbacks, including very poor solubility in water and a lack of tumor specificity. Herein, we report our development of the anticancer codrug **1**. This compound contains LENA (**2**, Relimid) and PTX (**4**, Taxol), both of which were connected to a sugar core bearing an enzyme recognition site.

The PTX molecule (**4**, C_47_H_51_NO_14_) is considerably bulkier than LENA (**2**, C_13_H_13_N_3_O_3_). Our initial approach was to attach PTX (**4**) to the least hindered primary hydroxyl group at the C5″ position of ribose **3**. As depicted in Scheme 2, (−)-d-ribose (**3**) was therefore converted to its benzaldehyde acetal **5**.^15^ To introduce a carbonate joint, we treated this intermediate with *p*-nitrophenyl chloroformate (**6**) in triethylamine (Et_3_N) and dichloromethane (CH_2_Cl_2_). The corresponding anomeric mixture of carbonates **7** was produced and then reacted with PTX (**4**) to give conjugates **8** in 80% yield. Repeat of this procedure by use of chloroformate **6** to react with conjugates **8** led to a mixture of bicarbonates **9** (α/β = 1:20) in a low yield (30%). After separation with high-performance liquid chromatography (HPLC), pure compound β-**9** was treated with LENA (**2**) in Et_3_N and *N*,*N*-dimethylformamide (DMF) under various conditions. Unfortunately, the desired PTX–ribose–LENA conjugate **10** was not obtained. Further attempts by use of activated LENA **11**, prepared from LENA (**2**) and chloroformate **6** as previously described, to couple with conjugates **8** also failed. These results indicate that the bulky PTX moiety in conjugate **8** impeded its coupling at the C1″ position with compound **11**, which also carried a bulky LENA moiety.

**Scheme 2:**
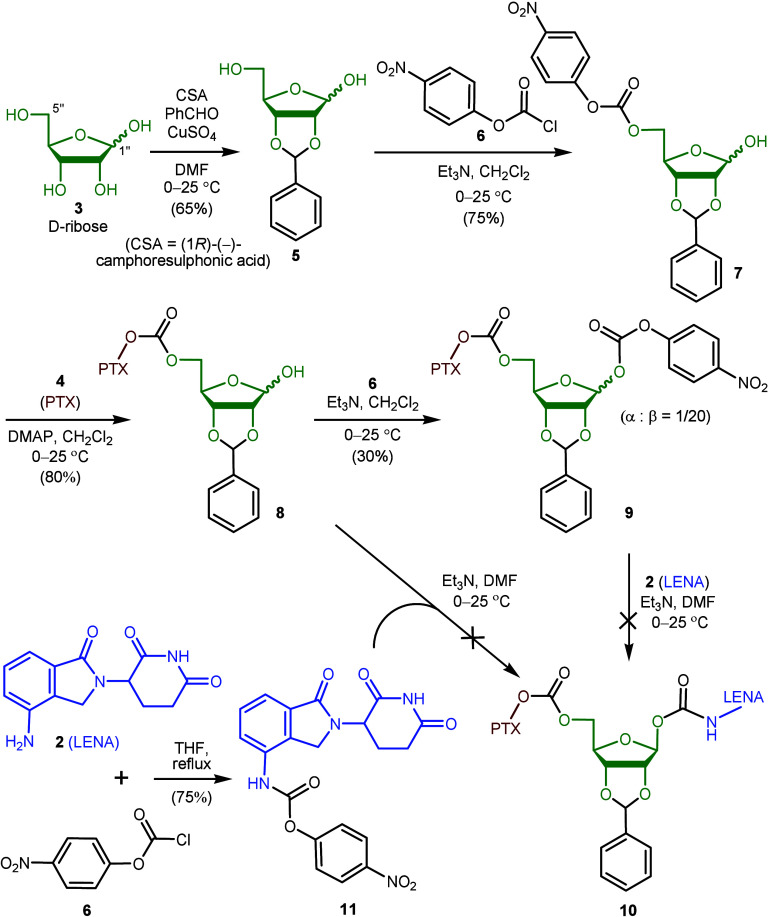
Attempt To Synthesize Codrugs Starting with PTX (**4**)

The second approach was to attach LENA (**2**) to the C5″ primary hydroxyl group of ribose **3** (Scheme 3). The *S* and *R* enantiomers of LENA are stable against racemization only at temperatures as low as −70 °C in buffer media. In neutral or alkaline solutions, these enantiomers undergo non-enzymatic racemization at 37 °C.^16^ Because LENA is commonly delivered at 25 °C, racemic LENA is often utilized in target syntheses involving acidic and basic reagents.

**Scheme 3:**
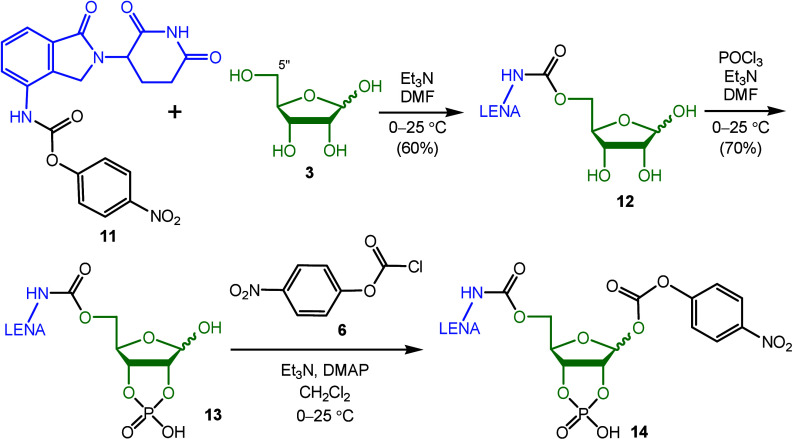
Attempt To Synthesize Codrugs Starting with LENA Derivative **11**

Coupling of activated LENA (**11**) with ribose **3** was performed in a solution of Et_3_N and DMF at room temperature. The resultant carbamates **12** were then phosphorylated with phosphorus(V) oxychloride (POCl_3_, 1.05 equiv) to give desired anomeric phosphodiesters **13** in 70% yield. Several attempts were made to add *p*-nitrophenyl chloroformate **6** onto the phosphodiesters **13**, but the desired coupling products **14** were not obtained. Instead, the major components detected in the reaction mixture were LENA (**2**) and a ribose derivative with a free hydroxyl group. This observation suggests that dephosphorylation occurred, followed by an intramolecular cyclization, leading to the release of LENA (**2**) from phosphodiesters **13***in situ*.

The successful synthesis of codrug **1** was accomplished by the third approach shown in Scheme 4. Therein, activated LENA (**11**) was conjugated with (−)-d-ribose benzaldehyde acetal **5** at its C5″ position in a solution of Et_3_N and DMF at room temperature. The resultant carbamates **15** were then treated with chloroformate **6** to give a mixture (α/β = 1:20) of carbonates **16** in 76% overall yield. After separation with HPLC, the desired β-anomer was treated with PTX (**4**) in 4-dimethylaminopyridine (DMAP) and CH_2_Cl_2_ to give pure β-carbonate **17** in 80% yield. PTX was then regioselectively attached through its C1″ hydroxyl group to the carbonate joint of compound **17**. This key step resolved the steric problem previously encountered during the conversion of compound **9** → compound **10**.

**Scheme 4:**
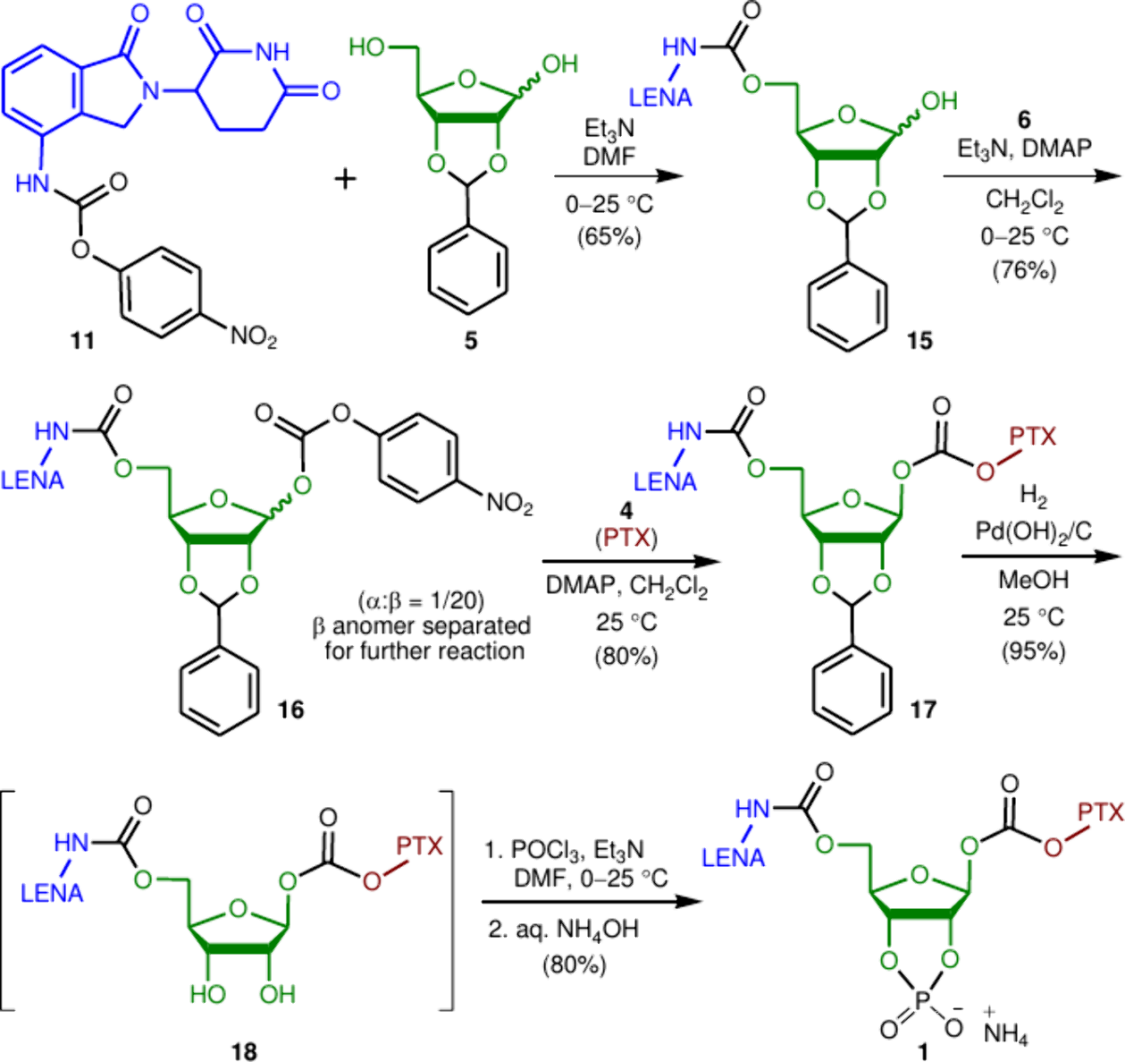
Successful Synthesis of Codrug **1** Containing Both LENA (**2**) and PTX (**4**)

Finally, we deprotected the benzylidene acetal moiety in compound **17** through hydrogenation using Pearlman’s catalyst [10 mol %, Pd(OH)_2_ on carbon] in methanol at 25 °C. Without isolation, resultant *cis*-diol **18** was phosphorylated with POCl_3_ (1.05 equiv) in the presence of Et_3_N and DMF. The reaction mixture was carefully quenched with ammonium hydroxide solution at 0 °C to afford the desired codrug **1** in 80% yield. The final step involved the introduction of a phosphate moiety onto conjugated target **1** as an enzyme recognition site. The structure identification of all new compounds shown in Schemes 2–4 was based on their high-resolution mass, ^1^H, ^13^C, and ^31^P nuclear magnetic resonance (NMR), and infrared (IR) spectroscopies. Detailed data for these compounds are provided in the Supporting Information.

The water solubility of codrug **1** was determined as 205.6 μg/mL at room temperature for 72 h by the shake-flask method.^17^ Afterward, the codrug degraded very slowly to fragments detected by their *m*/*z* values in a tandem mass spectrometry (MS/MS) spectrometer. In comparison to the parent PTX (**4**) with water solubility of 0.3 μg/mL,^18^ the solubility of PTX-containing codrug **1** was increased 685 times.

To release the two constituent drugs from codrug **1** as shown in Scheme 1, we treated codrug **1** (2.02 × 10^–4^ M in ammonium acetate buffer, pH 6.5, 20.0 μL) with phosphodiesterase^19^ (20 units) in a sodium pyrophosphate buffer (pH 6.5, 1.0 mL) at room temperature. It resulted in sequential release of PTX (**4**) and LENA (**2**), which were detected by HPLC (see Figure 1 and Table S1 of the Supporting Information). The half-life (*t*_1/2_) for the generation of PTX (**4**) and LENA (**2**) was 23.8 and 26.3 h, respectively. After 48 h, codrug **1** was completely hydrolyzed to give d-ribofuranose (**3**) via biscarbonate intermediate **22**. The presence of intermediate **22** was confirmed by a HPLC–mass spectrometer as 202.0112 for M^+^, which is very close to its theoretical value of 202.0114 for C_7_H_6_O_7_, as shown in the Supporting Information.

**Figure 1:**
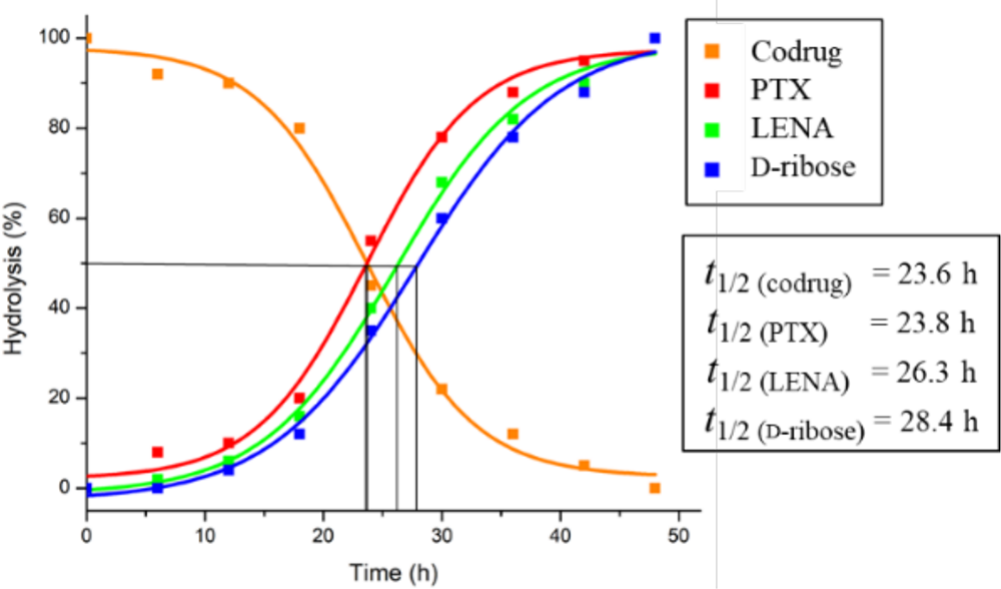
Hydrolysis of codrug **1** by phosphodiesterase in a sodium pyrophosphate buffer (pH 6.5) to generate LENA (**2**), PTX (**4**), and d-ribofuranose (**3**) at room temperature.

When phosphodiesterase reacted with water and codrug **1**, the C2″O–P bond was initially cleaved as illustrated in compound **19**.^20^ The resultant C2″–O^–^ group of compound **20** then attacked the carbonate joint at the C1″ position to release a PTX (**4**) molecule. As a result, a five-membered 1,3-dioxolan-2-one moiety was formed in the intermediate **21**.^21^ Subsequently, the C3″O–P bond therein was cleaved by phosphodiesterase, and the resultant C3″–O^–^ group attacked the carbamate joint at the C″5 position. This led to the release of the second drug, lenalidomide (**2**), and the formation of a six-membered 1,3-dioxan-2-one moiety in biscarbonate **22**.^21^ Hydrolysis of this intermediate **22** by water produced d-ribose **3**.^22^ The mechanistic pathway, described in Scheme 5, explains why the *t*_1/2_ value for PTX (**4**) was slightly shorter than that for LENA (**2**), as shown in Figure 1.

**Scheme 5:**
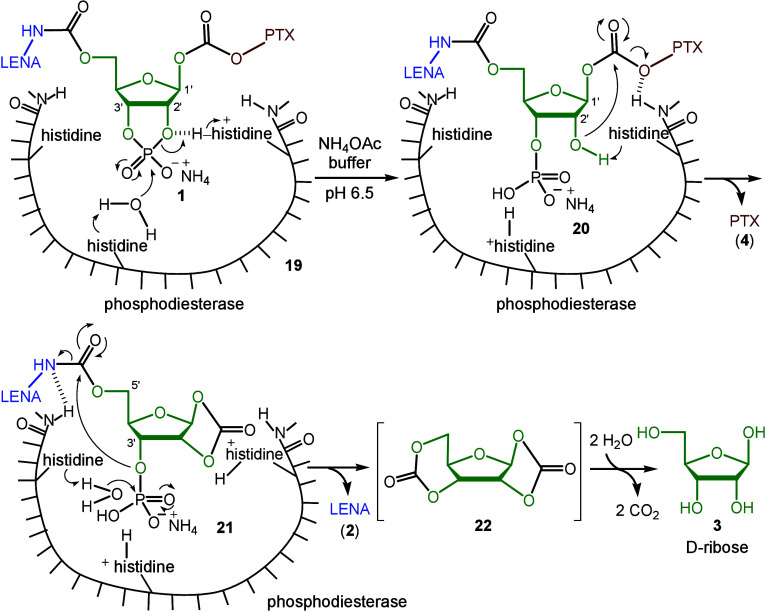
Mechanism on Release of LENA (**2**), PTX (**4**), and d-Ribose (**3**) from Codrug **1** Catalyzed by Phosphodiesterase

β-d-Ribofuranose is developed as an intriguing core moiety in conjunction with a phosphate moiety for the selective release of two drugs in codrug development. Codrug **1** is designed to contain four crucial components: LENA (**2**) as an immunomodulatory drug, PTX (**4**) as a potent anticancer drug, d-ribofuranose as a non-toxic core, and a phosphodiester group that acts as an enzyme recognition trigger. The synthesis of codrug **1** involves seven steps with a 33% overall yield. The inclusion of the phosphodiester moiety may enhance selectivity for cancer cells over normal cells. Moreover, this current codrug strategy significantly improves the water solubility of PTX (**4**) by more than 2 orders of magnitude.

## Data Availability

The data underlying this study are available in the published article and its online Supporting Information.

## Supporting Information Available

Materials, experimental details, spectroscopic data of all new compounds, structural identification of the target codrug **1**, references, and spectra (PDF)
