# Supplementary material for: β-d-Ribofuranose as a Core with a Phosphodiester Moiety as the Enzyme Recognition Site for Codrug Development
Source: Org Lett. 2024 Nov 14;26(46):9865–70. doi: 10.1021/acs.orglett.4c03662 (PMC11590094; doi:10.1021/acs.orglett.4c03662)
Supplement: Supplementary file 1 — ol4c03662_si_001.pdf [file ol4c03662_si_001.pdf]

Supporting Information

β-D-Ribofuranose as a Core with Phosphodiester Moiety as Enzyme Recognition Site for Codrug Development

Jih Ru Hwu,[a,*] Avijit Panja,[a] Shwu-Chen Tsay,[a] Wen-Chieh Huang,[a,b] Shu-Yu Lin,[b] Chen-Sheng Yeh,[c] Wu-Chou Su,[d] Li-Xing Yang,[e] and Dar-Bin Shieh[e,*]

[a]Department of Chemistry & Frontier Research Center on Fundamental and Applied Sciences of Matters, National Tsing Hua University, Hsinchu 300, Taiwan

[b]Institute of Biotechnology and Pharmaceutical Research, National Health Research Institutes, Miaoli County 350401, Taiwan

[c]Department of Chemistry, National Cheng Kung University, Tainan 701, Taiwan

[d]Department of Internal Medicine, National Cheng Kung University, Tainan 701, Taiwan

[e]Department of Dentistry and Institute of Oral Medicine, National Cheng Kung University, Tainan 701, Taiwan

Table of Contents

General Information S2

Experimental Procedures for the Production of New Compounds S3

Identification of the Structure of Codrug 1 .... S9

References S10

Spectra of New Compounds S12

HPLC-Mass Spectrum Containing Compound 22 .... S21

HPLC Chromatograms for Detection of the Sequential Release of Pacitaxel (4) and Lenalidomide (2) from Codrug 1 .... S22

General Information

All reactions were carried out in oven-dried glassware (120 °C) under an atmosphere of nitrogen unless as indicated otherwise. Carbon tetrachloride (CCl_4), *N,N*-dimethylformamide (DMF), and methanol (MeOH) were purchased from Mallinckrodt Chemical Co. Acetonitrile (MeCN), dichloromethane (CH_2Cl_2), ethyl acetate (EtOAc), hexanes, and toluene ($PhCH_3$) from Mallinckrodt Chemical Co. were dried and distilled from CaH_2. Tetrahydrofuran (THF) from Mallinckrodt Chemicals Co. was dried by distillation from sodium and benzophenone under N_2 atmosphere. The reagents purchased from Alfa Aesar included benzaldehyde (PhCHO), *N,N'*-dimethylpyridine-4-amine (DMAP), 10% palladium hydroxide on charcoal ($Pd(OH)_2/C$), and phosphorus oxychloride ($POCl_3$). The reagents purchased from Sigma-Aldrich included 2,2′-azobis(2-methylpropionitrile) (AIBN), *N*-bromosuccinamide (NBS), 1,1′-carbonyldiimidazole (CDI), lenalidomide, *p*-nitrophenyl chloroformate, (−)-paclitaxel, D-(−)-ribose, and triethylamine (Et_3N). Ammonium hydroxide (NH_4OH), (*R*)-(−)-10-camphoresulphonic acid (CSA), copper sulphate ($CuSO_4$) and, potassium hydrogenosulfate ($KHSO_4$) were purchased from Tokyo Chemical Industry Co.

Analytical thin layer chromatography (TLC) was performed on precoated plates (silica gel 60 F-254). Purification by gravity column chromatography was carried out by use of Silicycle ultra-pure silica gel (particle size 40–63 μM, 230–400 mesh). HPLC analysis was performed on high performance liquid chromatography with UV detection monitored at 254 nm by use of Chiralcel OD-H (250 mm × 20 mm i.d.) column with water and MeCN as the eluent.

Infrared spectra (IR) were measured on a Fourier transform infrared spectrometer (FT-IR). Absorption intensities are recorded by the following abbreviations: s, strong; m, medium; and w, weak. Proton NMR spectra were obtained on a 400 MHz spectrometer. Carbon-13 NMR spectra

were obtained on a 101 MHz spectrometer. For ^{1}H and ^{13}C spectra, calibration was made by use of the residual signals of partially deuterated solvents (i.e., CD_3OD, $CDCl_3$, and DMSO-d_6) reported by Fulmer *et al.* in 2010.[1] Chemical shifts (δ) are reported in ppm relative to the residual solvents $CDCl_3$ signal (δ = 7.24 for ^{1}H NMR and δ = 77.0 for ^{13}C NMR), CD_3OD signal (δ = 3.33 for ^{1}H NMR), DMSO-d_6 signal (δ = 39.5 for ^{13}C NMR), and H_3PO_4 used as external standard (δ = 0.00 for ^{31}P NMR). Multiplicities are recorded by the following abbreviations: s, singlet; d, doublet; dd, doublet of doublet; t, triplet; q, quartet; m, multiplet; and *J*, coupling constant (hertz). High-resolution mass spectra (HRMS) were measured on an instrument using a time-of-flight mass analyzer (TOF) with electrospray ionization (ESI).

Experimental Procedures for the Production of New Compounds

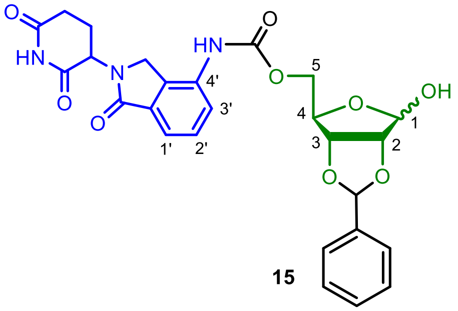

15

2,3-*O*-Benzylidene-5-*O*-[(lenalidomid-4'-*N*-yl)carbonyl]-β-D-ribofuranose (15). To a solution of benzaldehyde acetal **5**[2] (201 mg, 0.844 mmol, 1.0 equiv) and activated lenalidomide **11**[3a] (376 mg, 0.886 mmol, 1.05 equiv) in DMF (3.0 mL) under N_2 atmosphere was added Et_3N (90.7 mg, 0.902 mmol, 1.02 equiv) at 0 °C.[3a] After the reaction mixture was stirred at 25 °C for 4.0 h, the reaction mixture was quenched with water (1.0 mL), filtered, and concentrated under reduced pressure to afford the crude product. It was then purified by use of column chromatography (5.0 % MeOH in CH_2Cl_2 as the eluent) to give the desired lenalidomide–ribose carbamates **15** (284 mg, 0.548 mmol) in 65% yield as white solids: TLC R_f 0.45 (10% MeOH in CH_2Cl_2 as the eluent);

mp (recrystallized from MeOH) 268.8–270.4 °C; ^{1}H NMR (CD_3OD, 400 MHz) δ 7.79 (d, J = 7.6 Hz , 1 H), 7.59 (t, J = 7.6 Hz, 1 H), 7.46–7.37 (m, 3 H), 7.36–7.35 (m, 3 H), 6.30 (s, 1 H), 5.81 (s, 1 H), 5.20–5.15 (m, 1 H), 4.90 (d, J = 6.2 Hz, 1 H), 4.77–4.62 (m, 2 H), 4.59 (d, J = 6.2 Hz, 1 H), 4.39–4.37 (m, 1 H), 4.17–4.10 (m, 2 H), 2.95–2.75 (m, 2 H), 2.36–2.21 (m, 1 H), 2.19–2.15 (m, 1 H); ^{13}C NMR (DMSO-d_6, 101 MHz) δ 172.9, 171.2, 171.0, 149.6, 135.2, 132.2, 129.2, 128.8, 128.1, 126.1, 125.9, 116.3, 115.8, 110.4, 107.9, 105.8, 89.1, 85.6, 81.9, 64.8, 51.5, 45.5, 31.7, 22.7; IR (neat) 3332 (s, OH), 2952 (w), 1735 (s, C=O), 1601 (m), 1508 (m), 1358 (m), 1249 (s), 749 (w) cm^{-1}; HRMS (ESI-TOF) m/z [M + Na]$^+$ calcd for $C_{26}H_{25}N_3O_9$ + Na 546.1488, found 546.1480.

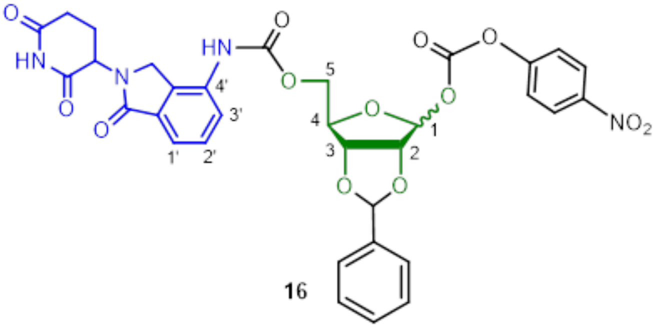

16

2,3-*O*-Benzylidene-5-*O*-[(lenalidomid-4'-*N*-yl)carbonyl]-1-*O*-[(*p*-nitrophenoxy)carbonyl]-D-ribofuranose (16). To a solution of lenalidomide–D-ribose carbamates **15** (105 mg, 0.201 mmol, 1.0 equiv) and *p*-nitrophenyl chloroformate **6** (41.2 mg, 0.205 mmol, 1.02 equiv) in CH_2Cl_2 (2.0 mL) under N_2 atmosphere was added DMAP (1.35 mg, 0.011 mmol, 0.05 equiv) and Et_3N (20.5 mg, 0.203 mmol, 1.01 equiv) at 0 °C.[3b] After the reaction mixture was stirred at 25 °C for 12 h, the reaction mixture was quenched with water (1.0 mL) and then washed with aq. $KHSO_4$ (3 × 10 mL) and then extracted with EtOAc (3 × 15 mL). The combined organic layers were dried over anhydrous $MgSO_4$, filtered, and concentrated under reduced pressure to afford the crude product. It was then purified by use of column chromatography (30% EtOAc in hexanes as the eluent) to

give the desired carbonate **16** (105 mg, 0.152 mmol) as a mixture (α:β = 1/20) in 76% overall yield, which was further purified by chiralcel OD-H column to give the desired β-isomer as greenish-white solids: TLC R_f 0.40 (5.0% MeOH in CH_2Cl_2 as the eluent); mp (recrystallized from MeOH) 272.6–274.8 °C; ^{1}H NMR (CD_3OD, 400 MHz) δ 8.14 (d, J = 8.4 Hz, 2 H), 7.80 (d, J = 7.6 Hz, 1 H), 7.62 (t, J = 7.6 Hz, 1 H), 7.58–7.47 (m, 3 H), 7.45–7.28 (m, 3 H), 6.83(d, J = 8.4 Hz, 2 H), 6.38 (s, 1 H), 5.96 (s, 1 H), 5.15–5.01 (m, 1 H), 4.80–4.70 (m, 1 H), 4.77–4.63 (m, 2 H), 4.51 (d, J = 6.2 Hz, 1 H), 4.42–4.37 (m, 1 H), 4.13–4.11 (m, 2 H), 2.91–2.69 (m, 2 H), 2.43–2.34 (m, 1 H), 2.18–2.14 (m, 1 H); ^{13}C NMR ($CDCl_3$, 101 MHz) δ 172.9, 171.2, 171.1, 155.4, 152.0, 149.6, 145.8, 135.3, 132.2, 130.0, 129.2, 128.8, 126.3, 126.1, 125.9, 122.1, 116.3, 115.8, 110.4, 107.9, 105.8, 89.7, 85.8, 81.9, 64.8, 51.5, 45.5, 31.8, 22.5; IR (neat) 2955 (w), 1738 (s, C=O), 1601 (m), 1535 (s, N–O), 1505 (s), 1358 (s, N–O), 1248 (s), 749 (w) cm^{-1}; HRMS (ESI-TOF) m/z [M + H]$^+$ calcd for $C_{33}H_{28}N_4O_{13}$ + H 689.1724, found 689.1725.

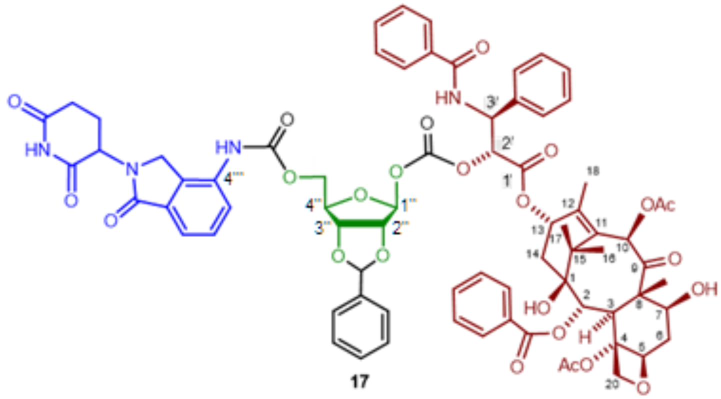

17

2'',3''-*O*-Benzylidene-5''-*O*-[(lenalidomid-4'''-*N*-yl)carbonyl]-1''-*O*-[(2'-paclitaxelyl)-carbonyl]-β-D-ribofuranose (17). To a solution of carbonate **16** (209 mg, 0.303 mmol, 1.0 equiv) and (–)-paclitaxel **4** (262 mg, 0.306 mmol, 1.01 equiv) in CH_2Cl_2 (3.0 mL) under N_2 atmosphere was added DMAP (37.7 mg, 0.309 mmol, 1.02 equiv) at 0 °C.[3c] After the reaction mixture was stirred at 25 °C for 48 h, the reaction was quenched with water (2.0 mL) and then extracted with

EtOAc (3 × 15 mL). The combined organic layers were dried over anhydrous $MgSO_4$, filtered, and concentrated under reduced pressure to afford the crude product. It was then purified by use of column chromatography (50% EtOAc in hexanes as the eluent) to give the desired lenalidomide–ribose–paclitaxel conjugate **17** (340 mg, 0.242 mmol) in 80% yield as white solids: TLC R_f 0.32 (5.0% MeOH in CH_2Cl_2 as the eluent); mp (recrystallized from MeOH) 292.2–294.4 °C; ^{1}H NMR ($CDCl_3$, 400 MHz) δ 8.10 (d, J = 7.6 Hz, 2 H), 7.70–7.62 (m, 2 H), 7.55–7.48 (m, 2 H), 7.47–7.40 (m, 4 H), 7.39–7.28 (m, 5 H), 7.27–7.13 (m, 8 H), 6.39 (s, 1 H), 6.30 (s, 1 H), 6.21 (t, J = 9.0 Hz, 1 H), 5.83 (s, 1 H), 5.79 (d, J = 2.8 Hz, 1 H), 5.62 (d, J = 7.2 Hz, 1 H), 5.48–5.44 (m, 1 H), 5.17–5.07 (m, 1 H), 4.93–4.80 (m, 2 H), 4.77–4.63 (m, 2 H), 4.44–4.21 (m, 5 H), 4.21–4.05 (m, 2 H), 3.79–3.64 (m, 1 H), 2.81–2.62 (m, 2 H), 2.51–2.40 (m, 1 H), 2.39 (s, 3 H), 2.37–2.26 (m, 2 H), 2.18 (s, 3 H), 2.15–2.08 (m, 2 H), 1.88–1.82 (m, 1 H), 1.77 (s, 3 H), 1.68 (s, 3 H), 1.23 (s, 3 H), 1.15 (s, 3 H); ^{13}C NMR ($CDCl_3$, 101 MHz) δ 203.7, 172.5, 171.1, 169.8, 169.7, 168.8, 167.4, 167.1, 166.9, 154.5, 149.8, 142.0, 136.7, 136.5, 133.5, 132.7, 132.6, 131.9, 131.5, 130.3, 130.2, 130.1, 129.1, 129.0, 128.9, 128.7, 128.6, 128.5, 128.3, 127.0, 126.4, 126.3, 122.4, 116.3, 115.8, 112.4, 110.2, 107.1, 88.6, 88.2, 85.1, 84.4, 81.5, 81.0, 79.9, 79.1, 75.5, 75.1, 72.0, 71.9, 65.3, 58.4, 52.5, 51.5, 45.4, 43.1, 35.5, 35.4, 29.6, 26.7, 22.6, 22.5, 22.2, 20.7, 14.7, 9.57; IR (neat) 3422 (s, OH), 2953 (m), 1735 (s, C=O), 1602 (m), 1506 (m), 1250 (s), 1171 (w), 749 (w) cm^{-1}; HRMS (ESI-TOF) m/z [M]$^+$ calcd for $C_{74}H_{74}N_4O_{24}$ 1402.4693, found 1402.4689.

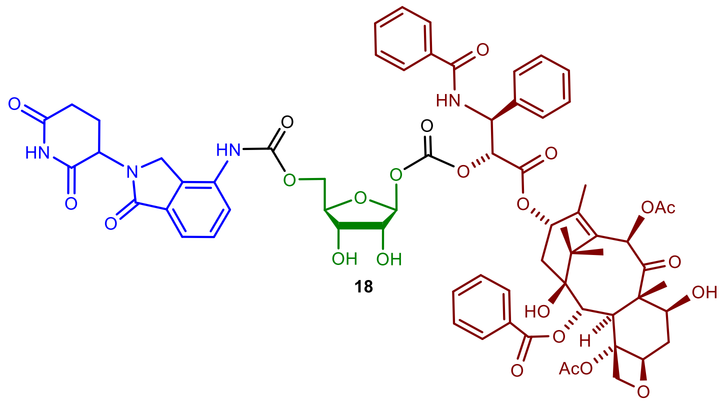

5''-*O*-[(Lenalidomid-4'''-*N*-yl)carbonyl]-1''-*O*-[(2'-paclitaxelyl)carbonyl]-β-D-ribofuranose (18). To a solution of benzylidene acetal **17** (152 mg, 0.108 mmol, 1.0 equiv) in MeOH (1.50 mL) under H_2 atmosphere (760 mm Hg) was added 10% $Pd(OH)_2/C$ (1.41 mg, 0.010 mmol, 0.10 equiv) at 25 °C.[3d,3e] After the reaction mixture was stirred at 25 °C for 1.0 h. The reaction mixture was filtered through a celite pad. Then the residue was dried under reduced pressure at 10–15 °C to afford the desired cis-diol conjugate **18** (134 mg, 0.102 mmol) in 95% yield as white solids. Subsequently, the resultant conjugate **18** was used to the next reaction without further purification: TLC R_f 0.30 (10% MeOH in CH_2Cl_2 as the eluent); HRMS (ESI-TOF) *m/z* $[M]^+$ calcd for $C_{67}H_{70}N_4O_{24}$ 1314.4380, found 1314.4383.

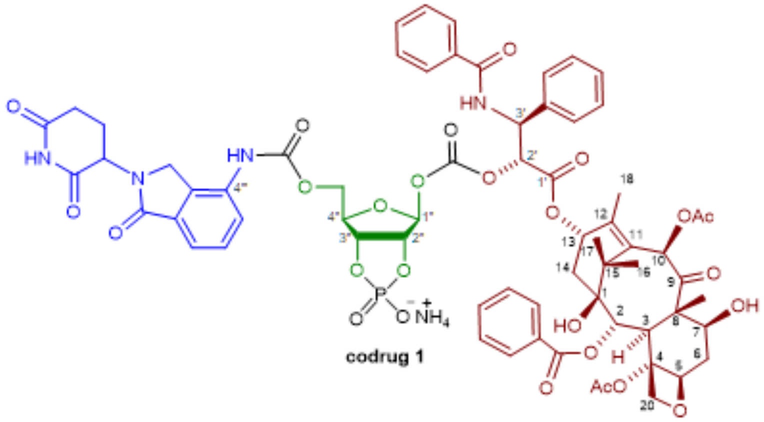

2'',3''-Cyclophosphate-5''-*O*-[(lenalidomid-4'''-*N*-yl)carbonyl]-1''-*O*-[(2'-paclitaxelyl)-carbonyl]-β-D-ribofuranose, Ammonium Salt (1). To a solution containing

lenalidomide–ribose–paclitaxel–cis-diol conjugate **18** (101 mg, 0.076 mmol, 1.0 equiv) and $POCl_3$ (12.3 mg, 0.081 mmol, 1.05 equiv) in DMF (1.0 mL) under N_2 atmosphere was added Et_3N (15.5 mg, 0.153 mmol, 2.02 equiv) at 0 °C.[3f] After the reaction mixture was stirred at 25 °C for 4.0 h, the reaction mixture was quenched with NH_4OH solution 1.0 M (82 μL) at 0 °C and then filtered through a celite pad and concentrated under reduced pressure to afford the crude product. It was then washed with dry EtOH (2 × 3.0 mL) to give the desired codrug **1** (85.1 mg, 0.061 mmol) in 80% yield as white solids: TLC R_f 0.21 (10% MeOH in CH_2Cl_2 as the eluent); mp (recrystallized from MeOH) 322.2–324.6 °C; ^{1}H NMR ($CDCl_3$, 400 MHz) δ 8.11 (d, J = 7.6 Hz, 2 H), 7.70–7.68 (m, 3 H), 7.57 (d, J = 7.2 Hz , 2 H), 7.50–7.40 (m, 5 H), 7.38–7.28 (m, 6 H), 6.31 (s, 1 H), 6.21 (s, 1 H), 6.17 (t, J = 9.0 Hz, 1 H), 5.79 (d, J = 2.8 Hz, 1 H), 5.62 (d, J = 7.2 Hz, 1 H), 5.45–5.42 (m, 1 H), 5.17–5.09 (m, 1 H), 4.92–4.86 (m, 1 H), 4.82 (d, J = 6.4 Hz, 1 H), 4.77 (d, J = 6.4 Hz, 1 H), 4.76–4.63 (m, 2 H), 4.40–4.21 (m, 4 H), 4.11–4.09 (m, 2 H), 3.75–3.63 (m, 1 H), 2.85–2.78 (m, 2 H), 2.62–2.48 (m, 1 H), 2.35 (s, 3 H), 2.32–2.22 (m, 2 H), 2.18 (s, 3 H), 2.15–2.10 (m, 2 H), 1.88–1.81 (m, 1 H), 1.77 (s, 3 H), 1.65 (s, 3 H), 1.21 (s, 3 H), 1.11 (s, 3 H); ^{13}C NMR ($CDCl_3$, 101 MHz) δ 203.5, 172.7, 171.2, 171.1, 170.3, 168.8, 167.7, 167.1, 166.9, 166.8, 154.0, 150.0, 142.0, 133.6, 132.6, 132.5, 131.9, 130.3, 130.1, 129.1, 129.0, 128.9, 128.7, 128.6, 128.5, 128.3, 127.0, 126.3, 122.4, 116.3, 115.9, 112.4, 110.2, 94.0, 92.1, 88.2, 84.3, 81.5, 80.9, 79.1, 75.5, 75.1, 72.0, 71.8, 65.2, 58.4, 52.5, 51.5, 45.5, 44.1, 43.1, 35.5, 35.4, 29.7, 26.6, 22.6, 22.5, 21.8, 20.7, 14.8, 9.60; ^{31}P NMR ($CDCl_3$, 162 MHz) δ 16.8; IR (neat) 3420 (s, OH), 2952 (w), 1735 (s, C=O), 1612 (m), 1501 (m), 1250 (s), 1050 (s, P=O), 554 (s, P–O) cm^{-1}; HRMS (ESI-TOF) m/z $[M]^+$ calcd for $C_{67}H_{72}N_5O_{26}P$ 1393.4203, found 1393.4205.

Identification of the Structure of Codrug 1

Its exact mass was measured as 1393.4205 for M^+, which is very close to its theoretical value of 1393.4203 for $C_{67}H_{72}N_5O_{26}P$. Among the 61 detected peaks in its ^{13}C NMR spectrum, eleven were in the downfield region at 203.5, 172.7, 171.2, 171.1, 170.3, 168.8, 167.7, 166.9, 166.8, 154.0, and 150.0 ppm. They were associated with the eleven C=O carbons, among which three came from the lenalidomide moiety, six came from the paclitaxel moiety, one came from the carbonate joint, and the last one came from the carbamate joint. In the upfield region, six peaks at 26.6 (C-17), 22.6 (C-4-CO_2CH_3), 21.8 (C-16), 20.7 (C-10-CO_2CH_3), 14.8 (C-18), and 9.60 (C-19) ppm correspond to the six CH_3 groups in the paclitaxel moiety.

The ^1H NMR spectrum of codrug **1** exhibited one singlet at 6.31 ppm without splitting, which corresponded to the C1" proton of ribose and confirmed the β configuration of the paclitaxel moiety. Two doublets at 4.82 and 4.77 ppm with J = 6.4 Hz corresponded to the two cis protons at the C3" and C2" positions of D-ribose, respectively. These protons shifted downfield by 1.11 ppm for the C3" proton and 1.22 ppm for the C2" proton in comparison with their original values (3.71 ppm for the C3" proton and 3.55 ppm for the C2" proton) in the parent ribose (**3**) due to their attachment to a phosphate group. A multiplet resonated at 4.1–4.09 ppm associated with the two C5" protons of ribose. These protons attached to a carbamate group were about 0.60 ppm downfield than the corresponding protons (3.51–3.48 ppm) attached to a hydroxyl group in the parent ribose. On the other hand, shift of the C2' proton of the parent paclitaxel from 4.77–4.75 ppm to 5.45–5.42 ppm as a multiplet in codrug **1** clearly indicates where the conjugation took place between ribose and paclitaxel through a carbonate joint. The six singlets at 2.35, 2.18, 1.77, 1.65, 1.21, and 1.11 ppm resulted from the six CH_3 groups in paclitaxel. Meanwhile, a multiplet between 5.17–5.09 ppm belonged to the only tertiary CH proton of the lenalidomide.

Finally, a ^{31}P NMR spectrum was obtained, in which one singlet was observed at 16.8 ppm relative to the peak of 85% phosphoric acid in D_2O at 0 ppm, which served as an external reference. Results from these spectra confirm our success on the installation of lenalidomide, paclitaxel, and a phosphate group on D-ribose in codrug **1**.

References

(1) Fulmer, G. R.; Miller, A. J. M.; Sherden, N. H.; Gottlieb, H. E.; Nudelman, A.; Stoltz, B. M.; Bercaw, J. E.; Goldberg, K. I. NMR chemical shifts of trace impurities: common laboratory solvents, organics, and gases in deuterated solvents relevant to the organometallic chemist. *Organometallics* **2010**, *29*, 2176–2179.

(2) Grindley, T. B.; Szarek, W. A. Configurational and conformational studies on some benzylidene derivatives of D-ribose and di-β-D-ribofuranose. 1,5':1',5-dianhydride *Carbohydr. Res.* **1972**, *25*, 187–195.

(3) Synthetic procedure used by a slight modification to prepare compounds **11** and **15**, see: (a) Chimmanamada, D. U.; Ying, W.-W. Targeted therapeutics. PCT Int. Appl. WO 2013/158644 A2, Oct 24, **2013**; (b) For compound **16**, see: Fomina, N.; McFearin, C.; Sermsakdi, M.; Edigin, O.; Almutairi, A. UV and near-IR triggered release from polymeric nanoparticles. *J. Am. Chem. Soc*. **2010**, *132*, 9540–9542. (c) For compound **17**, see: de Groot, F. M. H.; van Berkom, L. W. A.; Scheeren, H. W. Synthesis and biological evaluation of 2'-carbamate-linked and 2'-carbonate-linked prodrugs of paclitaxel: selective activation by the tumor-associated protease plasmin. *J. Med. Chem.* **2000,** *43,* 3093–3102. (d) For compound **18**, see: Zhang, C.; Liu, J.; Du, Y. Total synthesis of ribisin A. *Tetrahedron Lett*. **2014**, *55*, 959–961; (e) Bouvier, E.; Thirot, S.; Schmidt F.; Monneret C.

First enzymatically activated Taxotere prodrugs designed for ADEPT and PMT. *Bioorg. Med. Chem.* **2004**, *12*, 969–977. (f) For codrug **1**, see: Pitsch, S.; Spinner, C.; Atsumi, K.; Ermert, P. Synthesis of some D-ribose phosphates synthese einiger phosphorsäure-ester der D-ribose. *Chimia* **1999**, *53*, 291–94.

Spectra of New Compounds

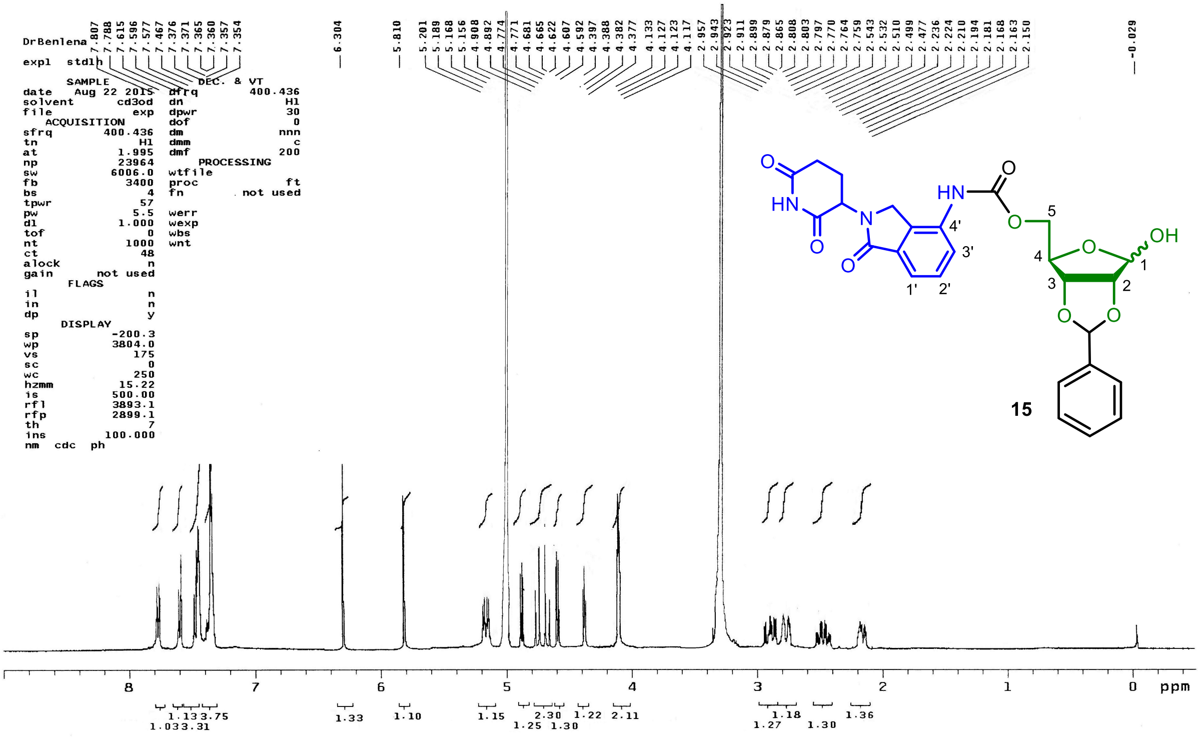

^{1}H NMR spectrum of compound **15** (400 MHz, CD_3OD)

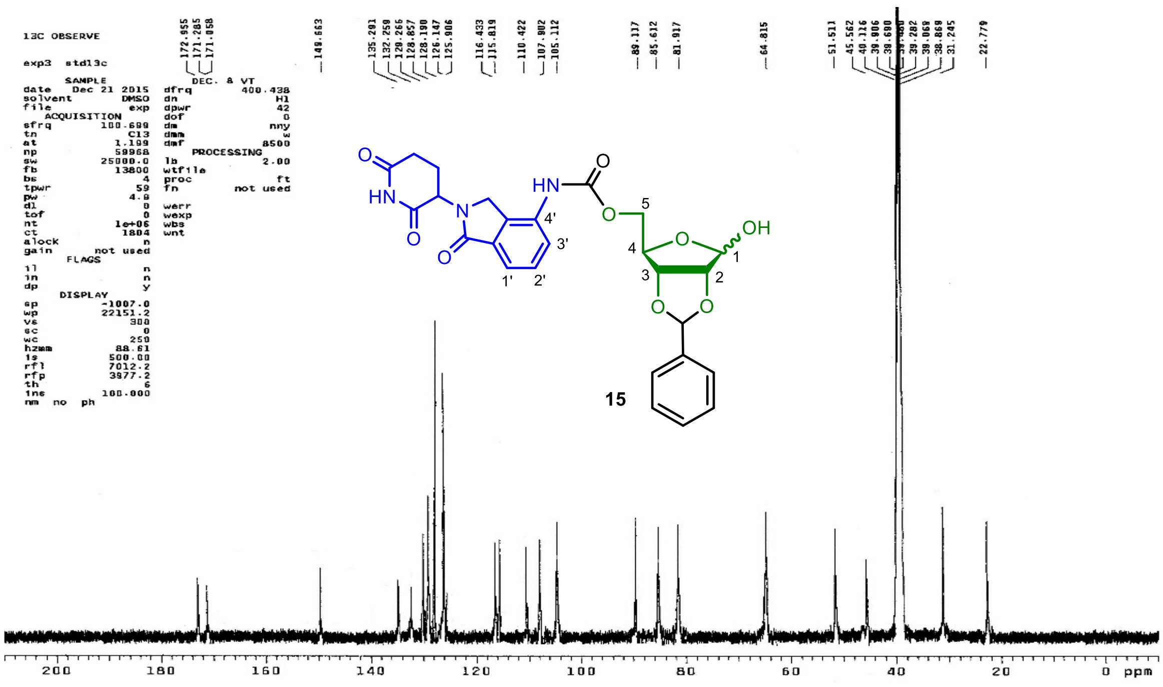

^{13}C NMR spectrum of compound **15** (101 MHz, DMSO-d_6)

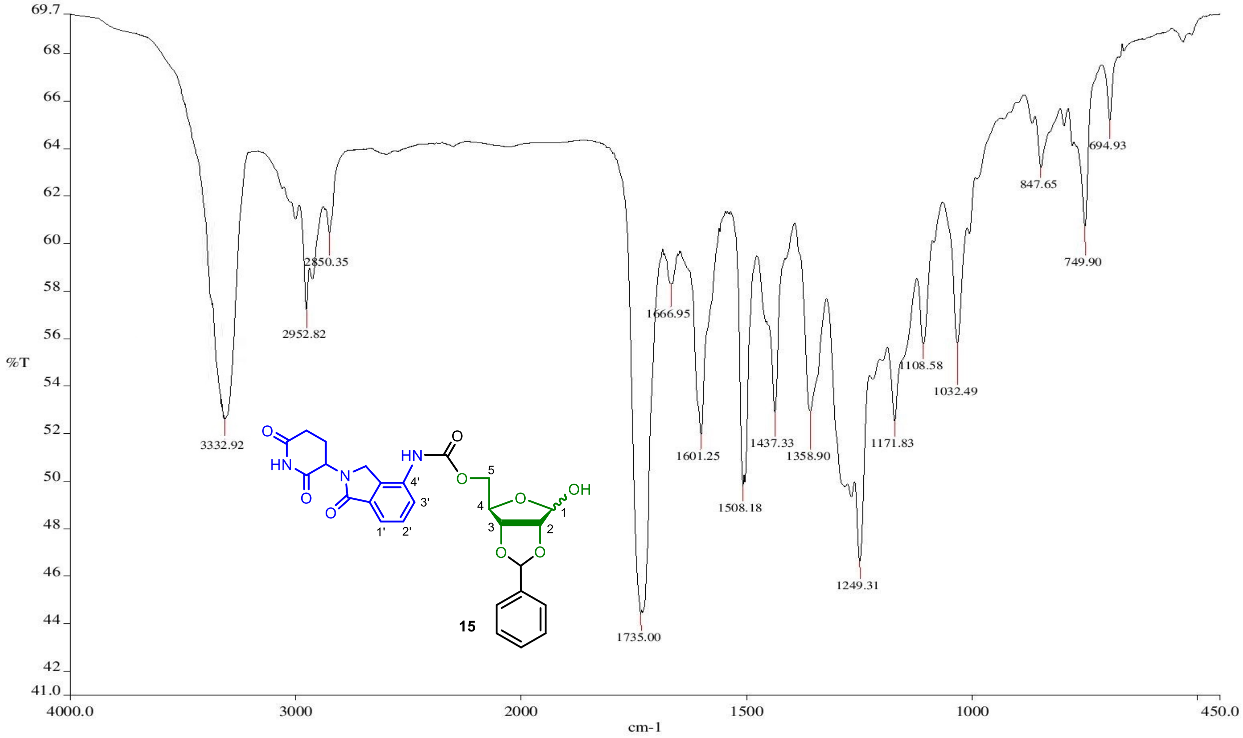

IR spectrum of compound **15**

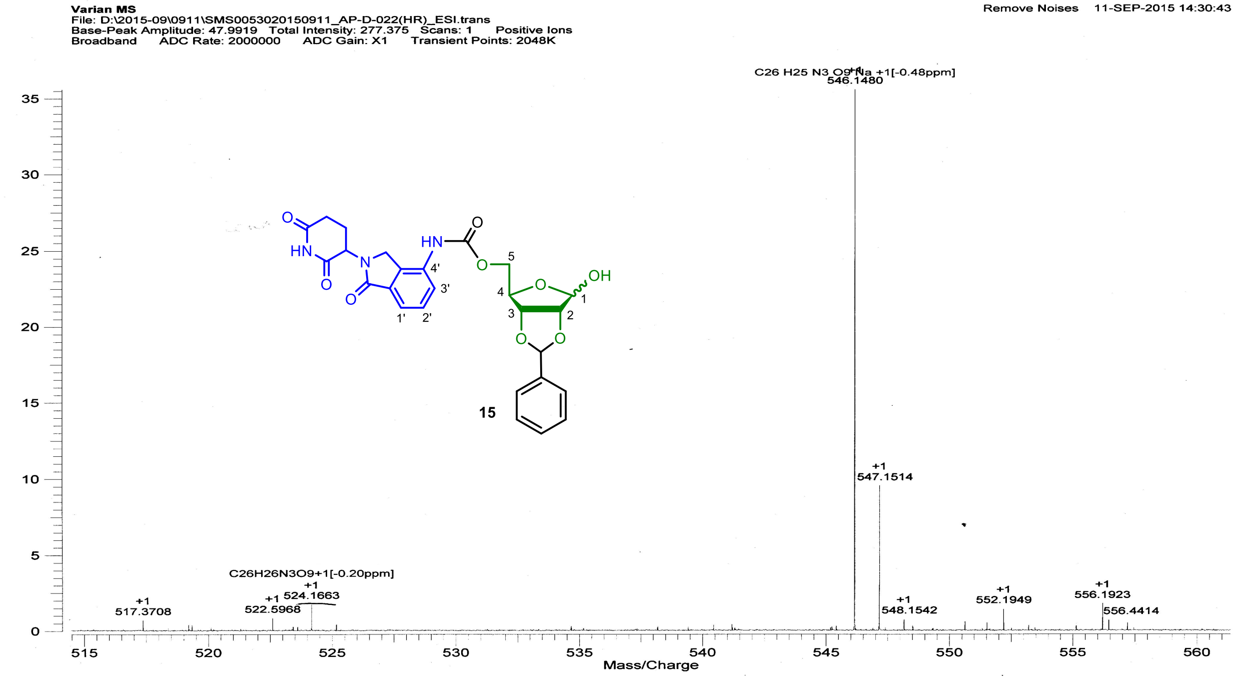

HRMS spectrum of compound **15**

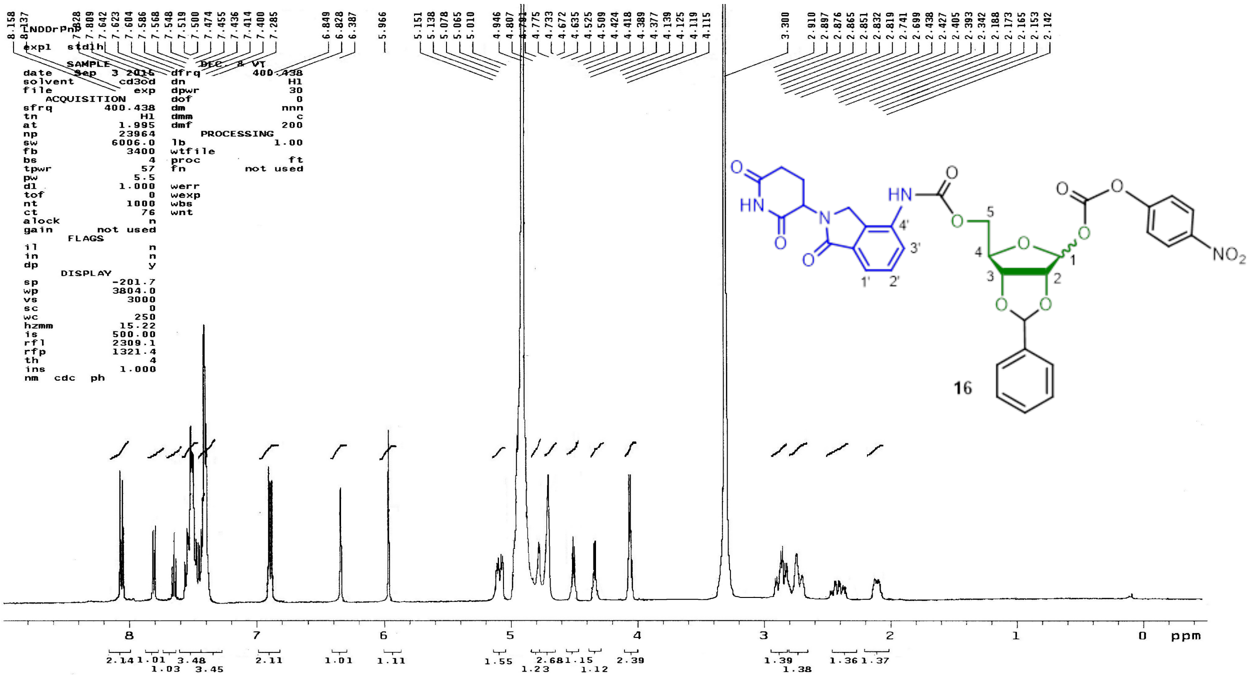

^{1}H NMR spectrum of compound **16** (400 MHz, CD_3OD)

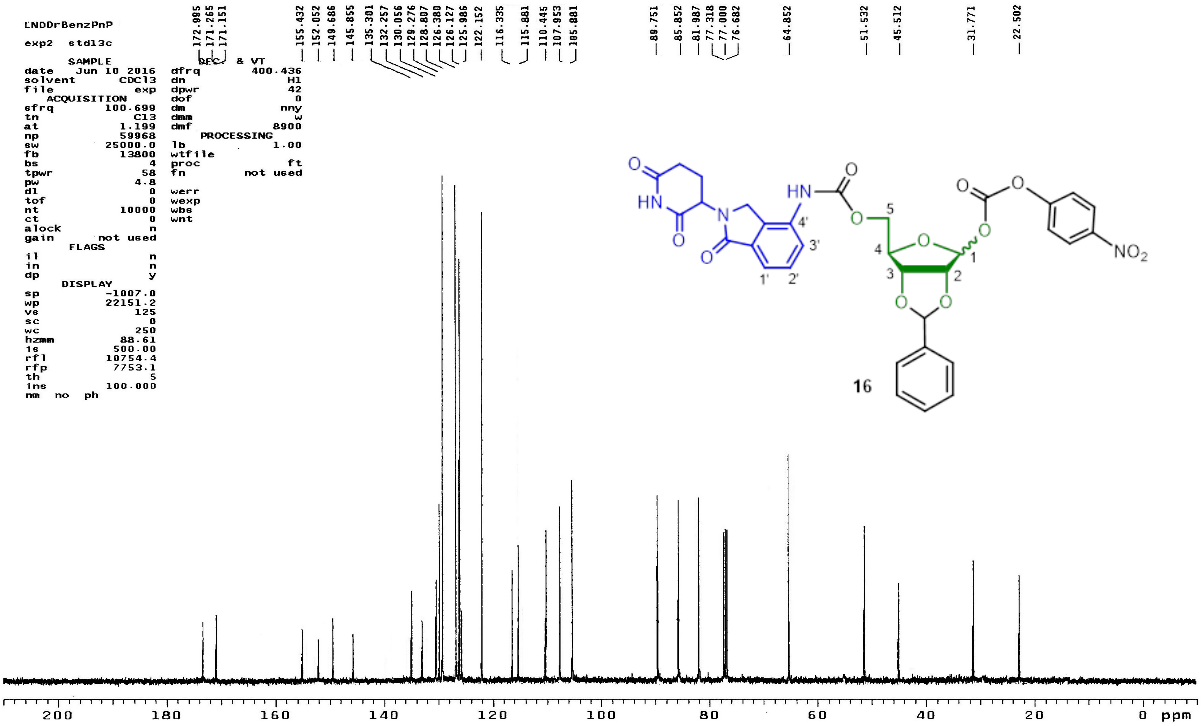

^{13}C NMR spectrum of compound **16** (101 MHz, $CDCl_3$)

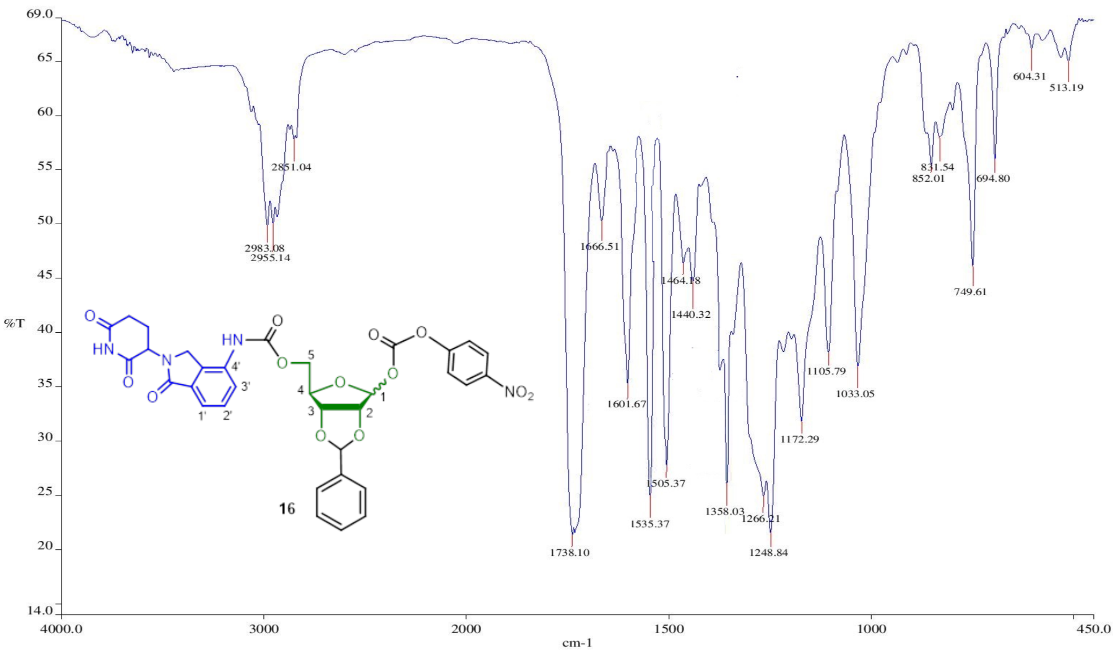

IR spectrum of compound **16**

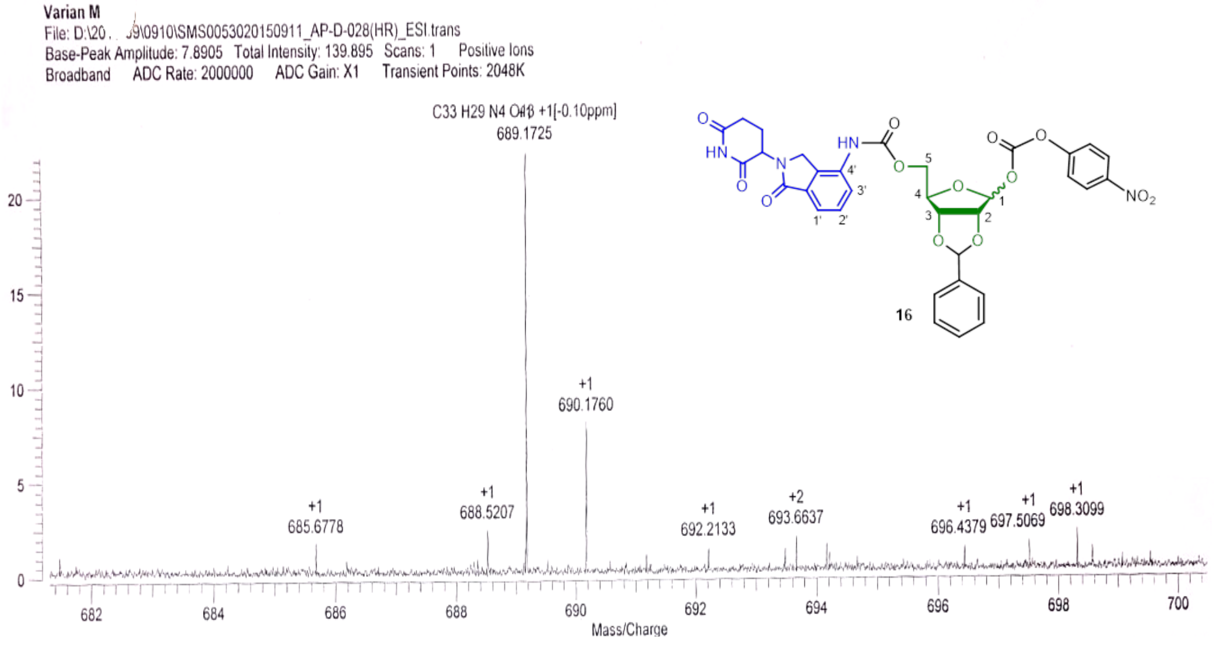

HRMS spectrum of compound **16**

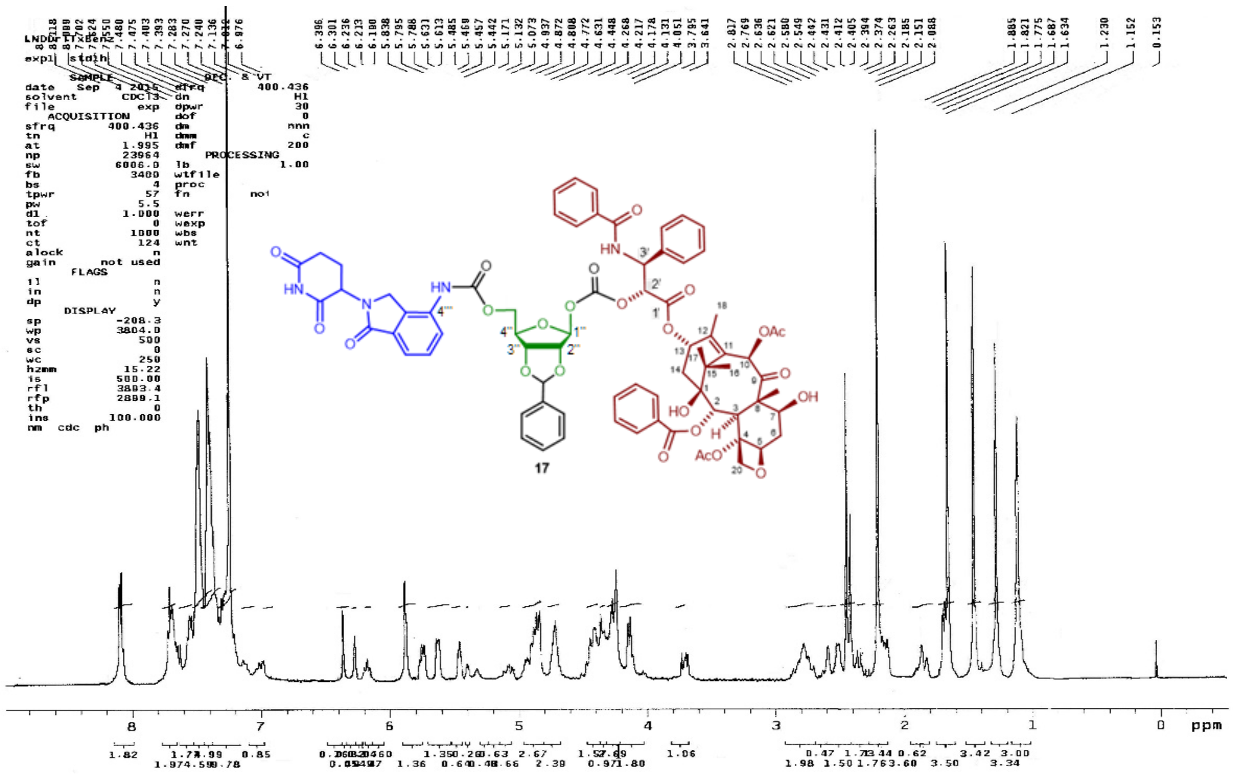

^{1}H NMR spectrum of compound **17** (400 MHz, $CDCl_3$)

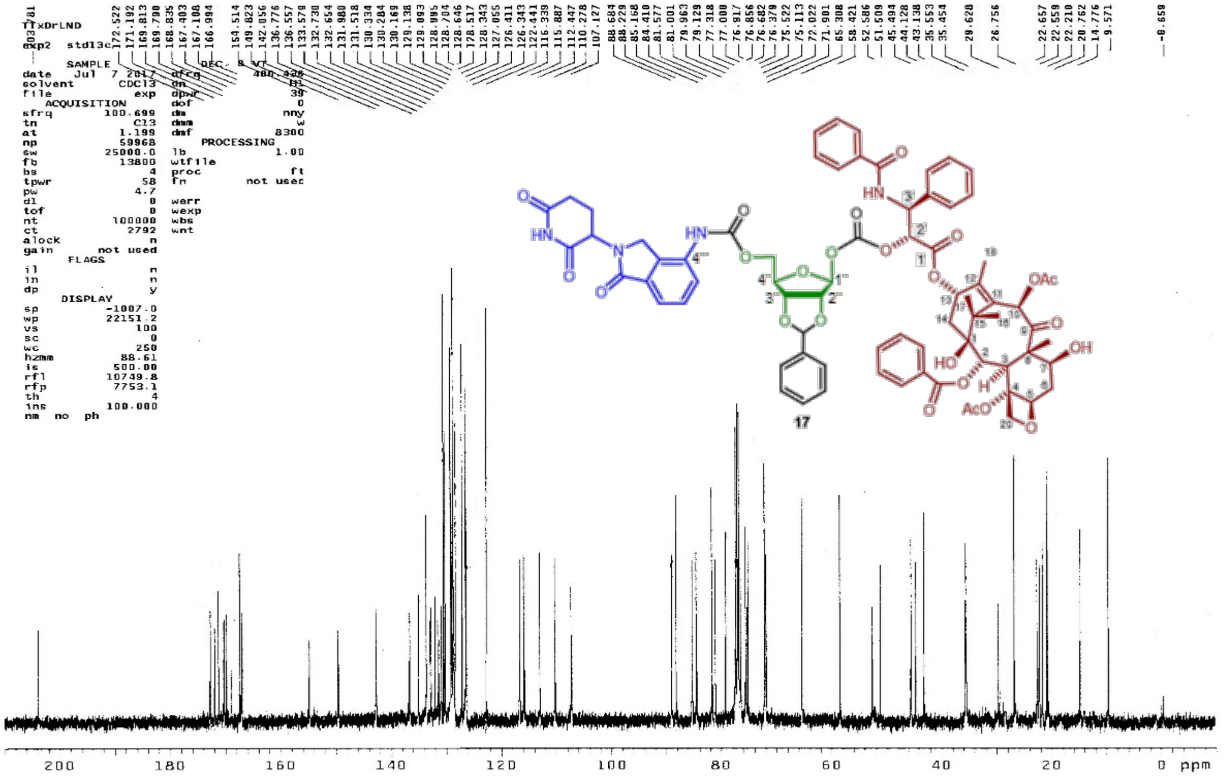

^{13}C NMR spectrum of compound **17** (101 MHz, $CDCl_3$)

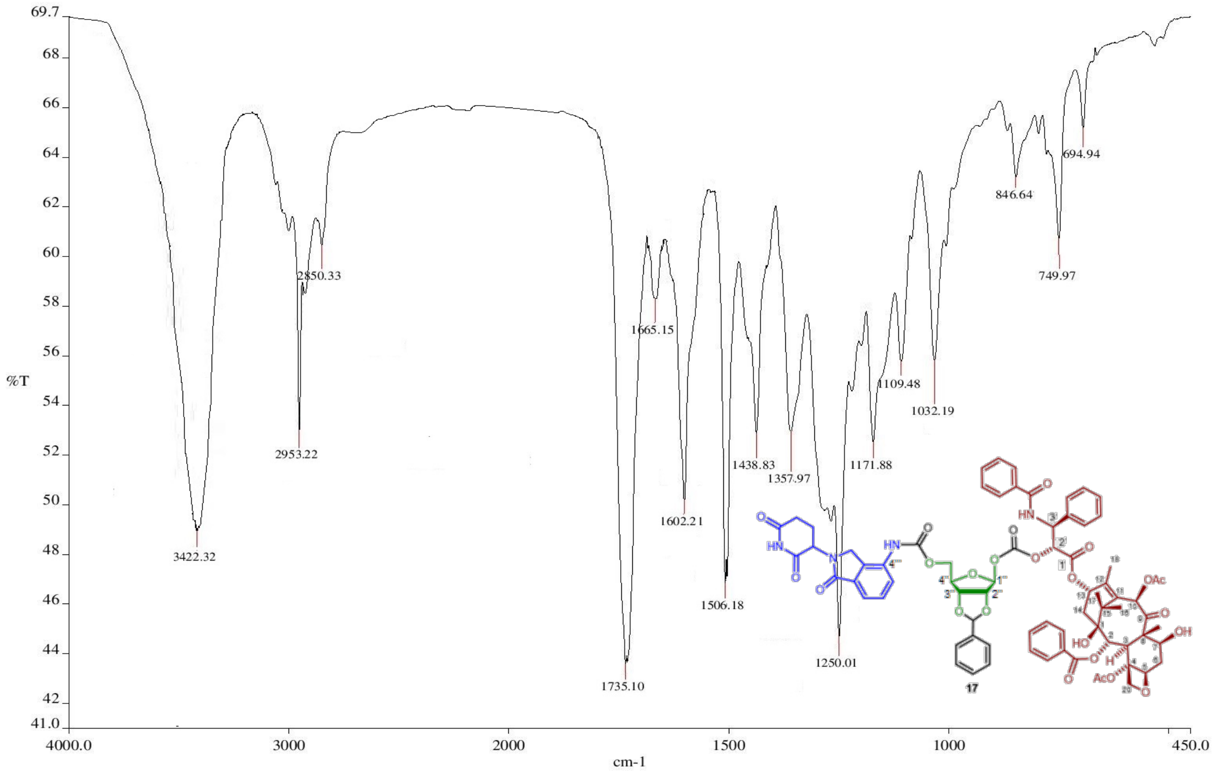

IR spectrum of compound **17**

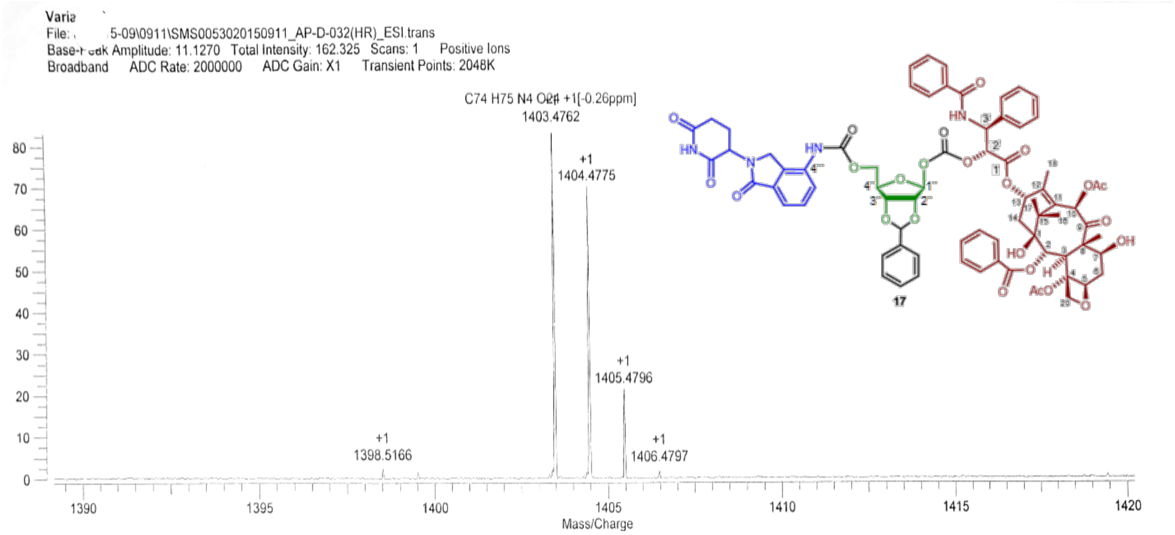

HRMS spectrum of compound **17**

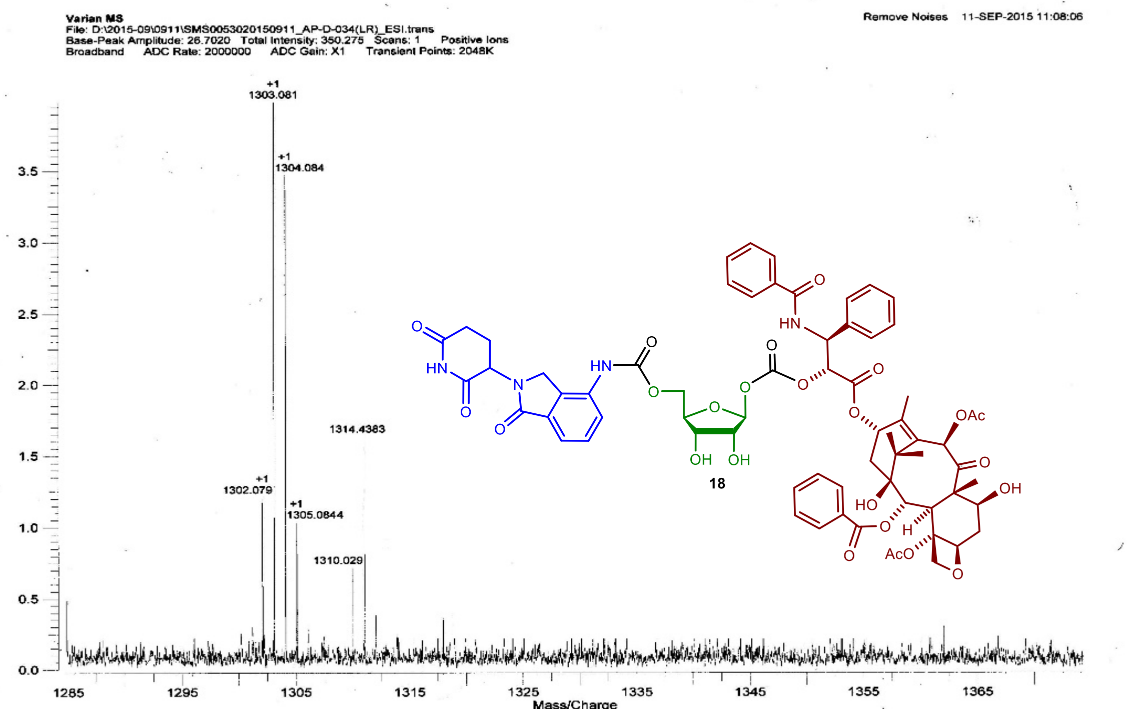

HRMS spectrum of compound **18**

Note: Due to instability of compound **18**, the ^{1}H NMR, ^{13}C NMR, and IR spectra are not available.

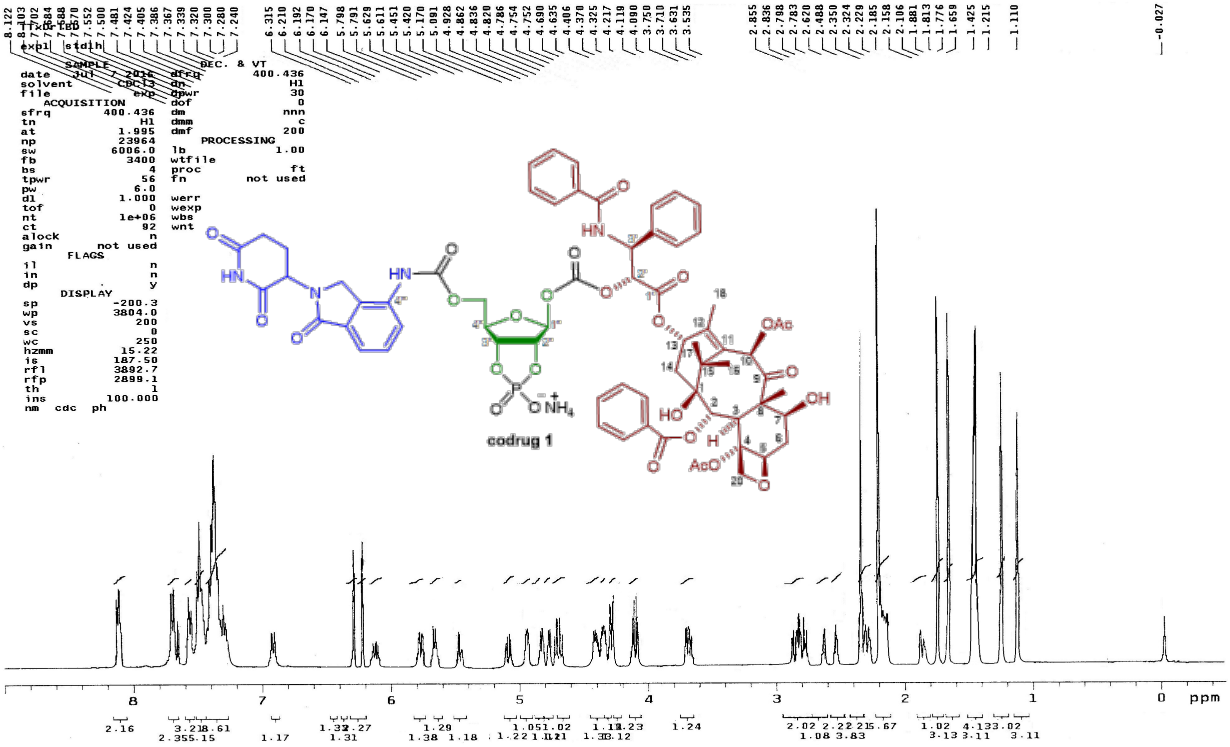

^{1}H NMR spectrum of codrug **1** (400 MHz, $CDCl_3$)

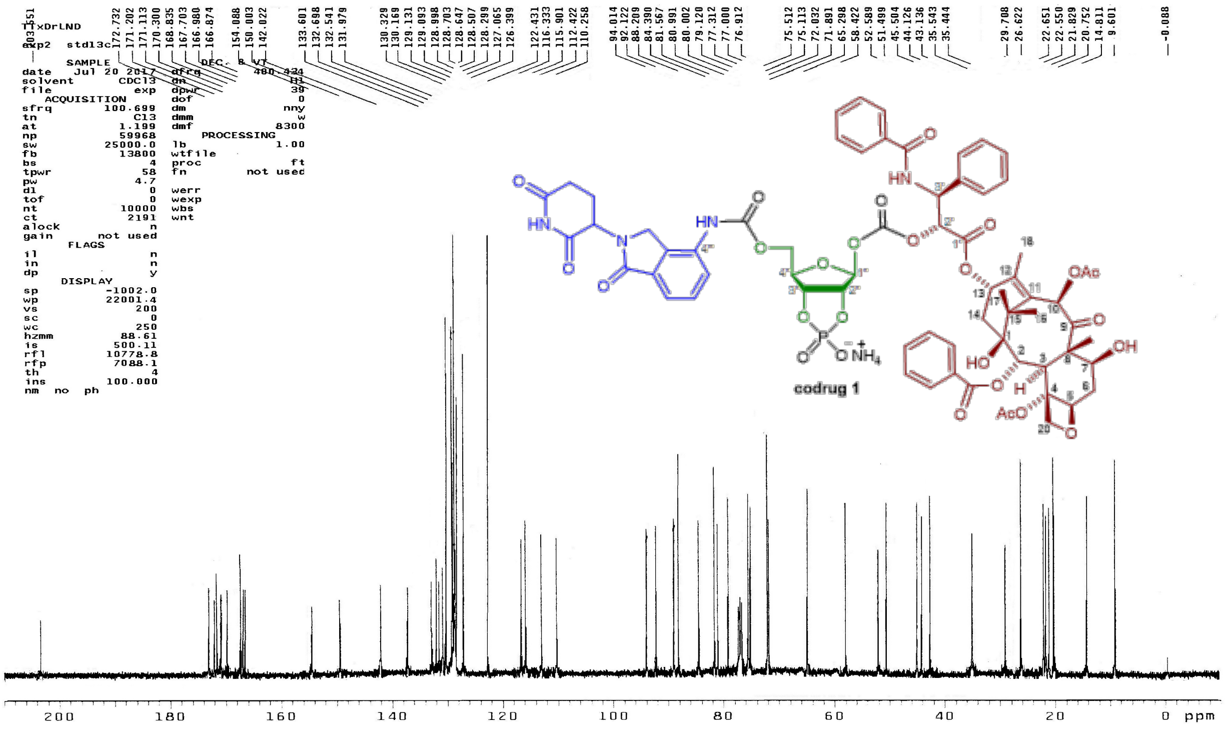

^{13}C NMR spectrum of codrug **1** (101 MHz, $CDCl_3$)

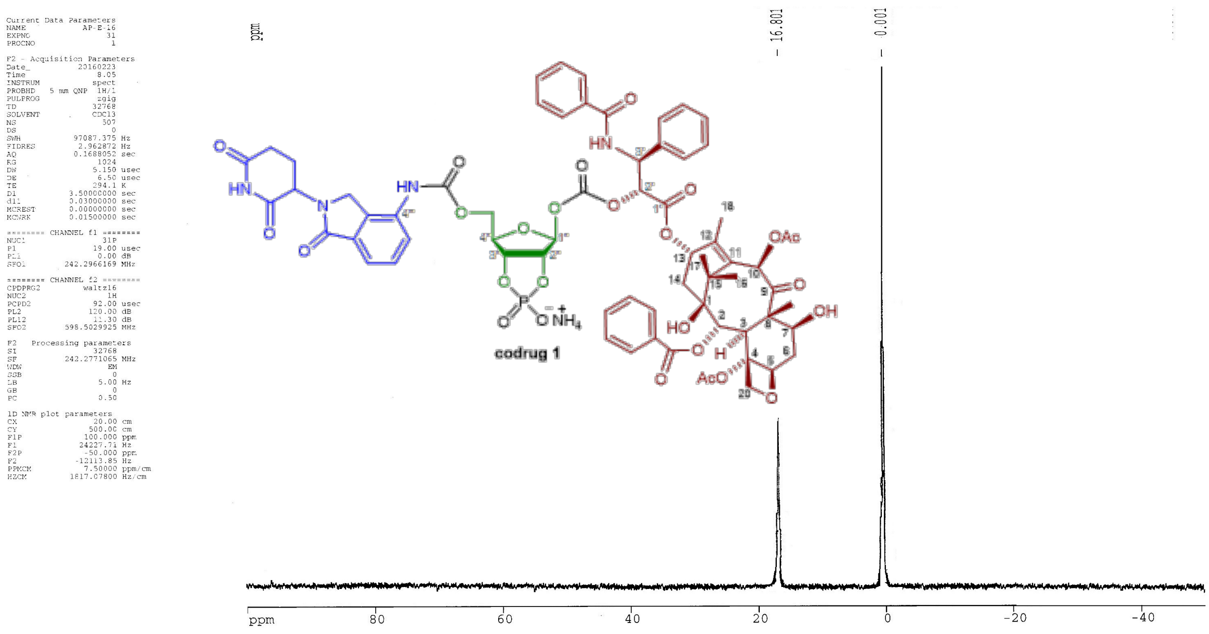

^{31}P NMR spectrum of codrug **1** (162 MHz, $CDCl_3$)

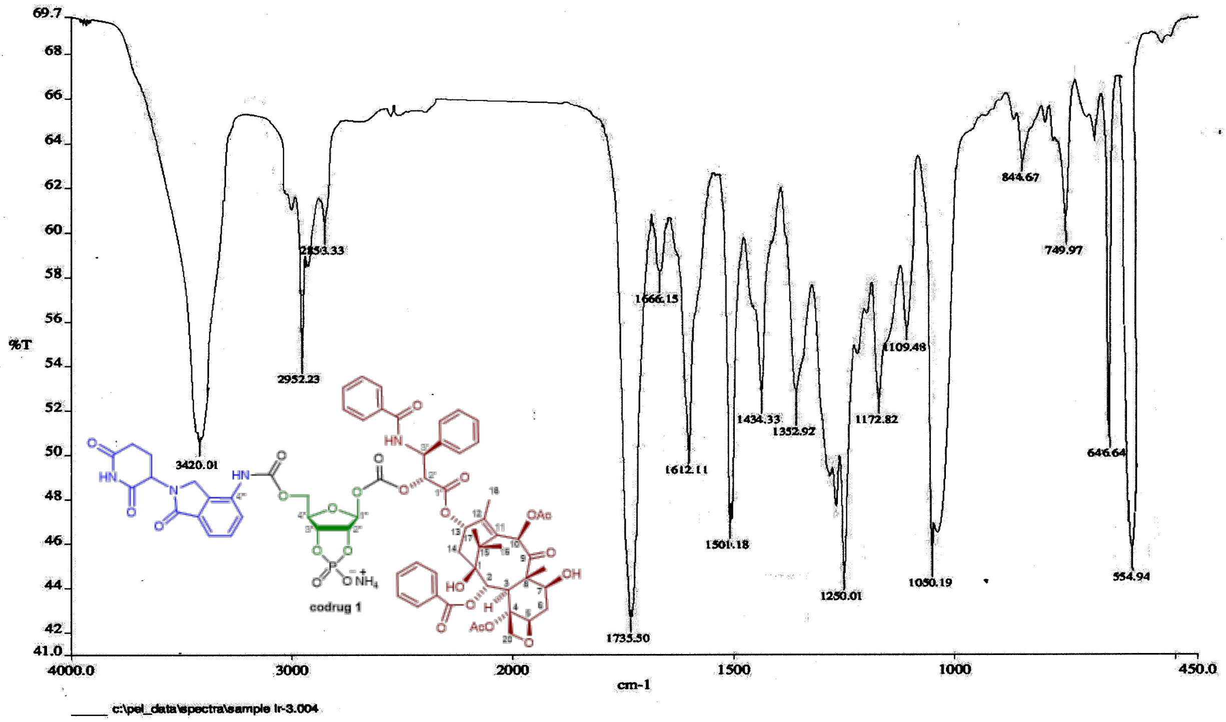

IR spectrum of codrug **1**

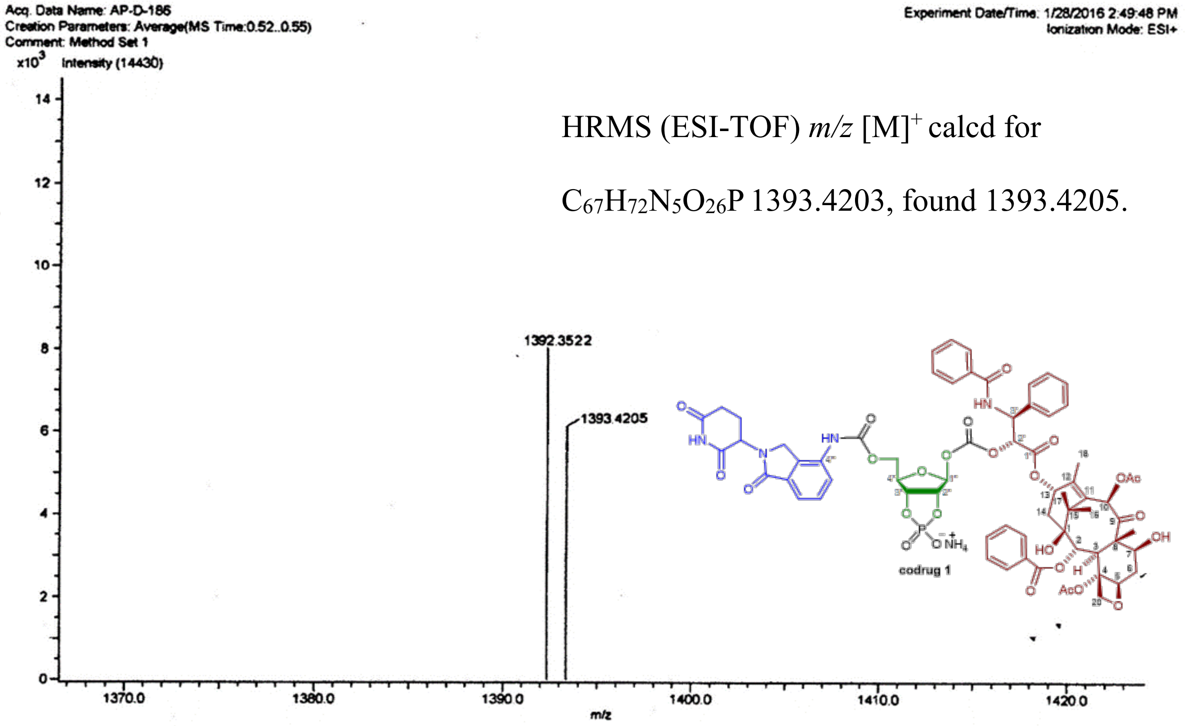

HRMS (ESI-TOF) *m/z* $[M]^+$ calcd for $C_{67}H_{72}N_5O_{26}P$ 1393.4203, found 1393.4205.

HRMS spectra of codrug **1**

HPLC-Mass Spectrum Containing Compound 22

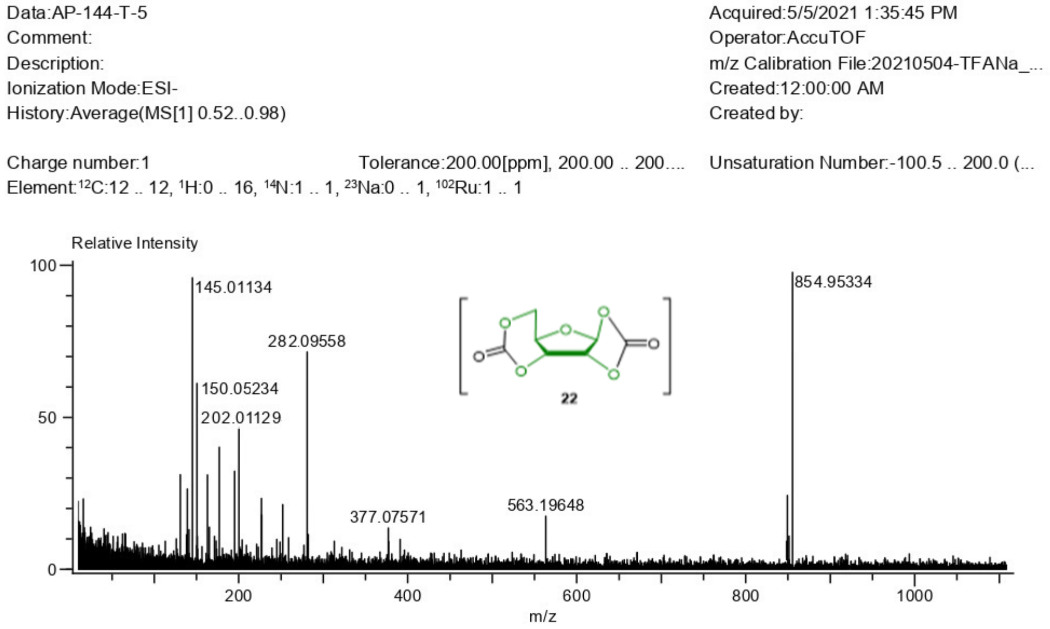

HRMS (ESI-TOF) *m/z* $[M]^+$ calcd for $C_7H_6O_7$ 202.0114, found 202.0112.

HPLC Chromatograms for Detection of the Sequential Release of Pacitaxel (4) and Lenalidomide (2) from Codrug 1

Mobile Phase: solvent A, Water (90%); solvent B, acetonitrile (10%).
UV Detector: λ = 254 nm.

Column: Thermo 5.0 μm Hypersil ODS (250 × 4.6 mm D.I.).

Flow rate: 1.0 mL/ min.

EChrom---REPORT

General Inforamtion

Analysis Date: 2019,02,14,11:41:04	Print Date: 2019,02,14
File Name:	
Operator: Administrator	

Experiment Condition

Material: OD-S	Flowrate: 1.0 ml/min
Mobile Phase:	Pressure: 10.0 MPa
Length: 250 mm	Detector: UV 254 nm
Diameter: 20mm	Volume: 20 米l

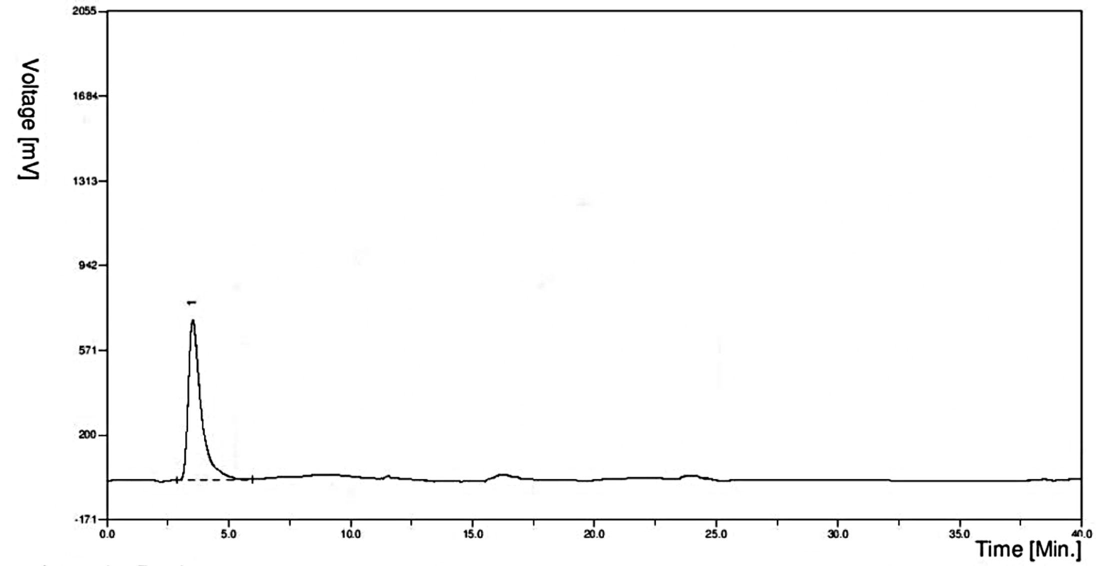

Integration Result

#	Name	Ret. Time(min)	Peak Height(mv)	Area(mv.sec)	Area Percentage(%)	Content(%)
1	Unknown	4.18	722.09	5221.74	100.000	0.0000
Total			722.09	5221.74	100	

HPLC chromatogram for hydrolysis of codrug **1** after 0 h

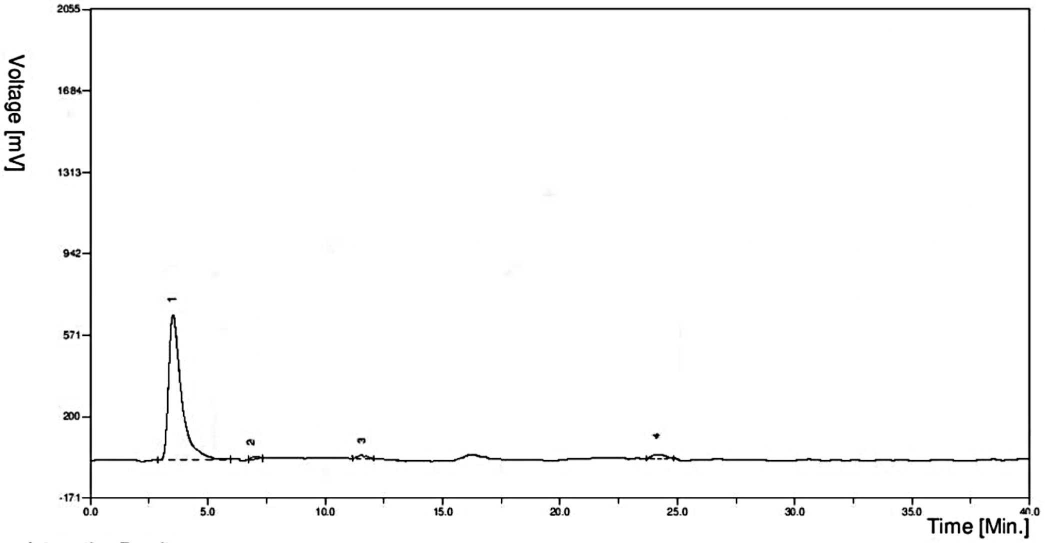

Integration Result

#	Name	Ret. Time(min)	Peak Height(mv)	Area(mv.sec)	Area Percentage(%)	Content(%)
1	Unknown	4.17	702.00	5064.24	96.280	0.0000
2	Unknown	7.07	1.35	19.35	0.200	0.0000
3	Unknown	12.01	2.45	50.49	0.960	0.0000
4	Unknown	24.17	5.01	134.65	2.560	0.0000
Total			710.81	5268.73	100	

HPLC chromatogram for hydrolysis of codrug **1** after 6.0 h

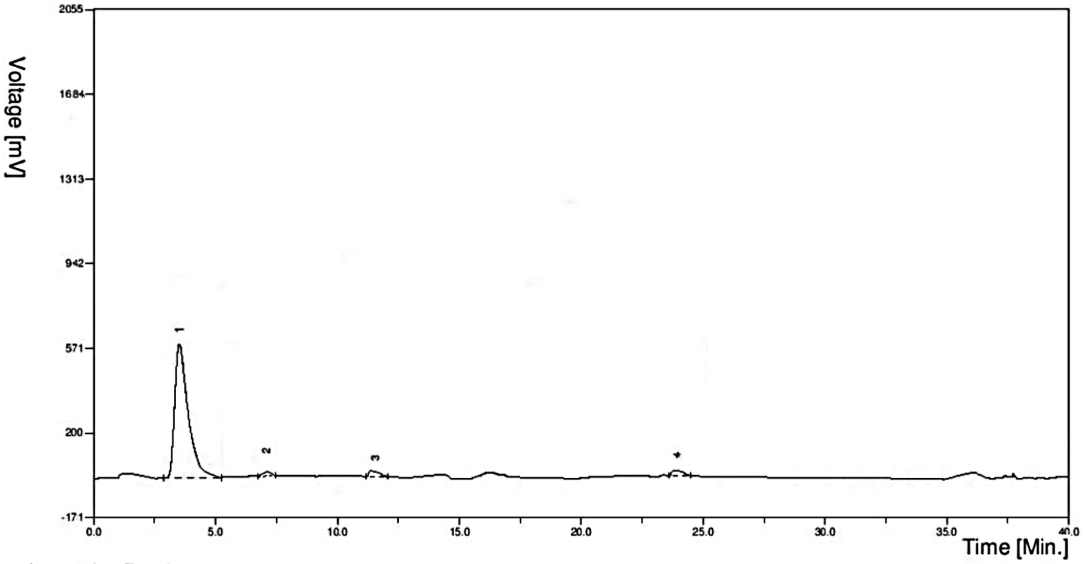

Integration Result

#	Name	Ret. Time(min)	Peak Height(mv)	Area(mv.sec)	Area Percentage(%)	Content(%)
1	Unknown	4.15	582.02	4106.14	93.120	0.0000
2	Unknown	7.05	2.95	35.35	0.802	0.0000
3	Unknown	12.02	6.21	126.98	2.880	0.0000
4	Unknown	24.15	5.01	134.65	3.198	0.0000
Total			596.19	4403.12	100	

HPLC chromatogram for hydrolysis of codrug **1** after 12 h

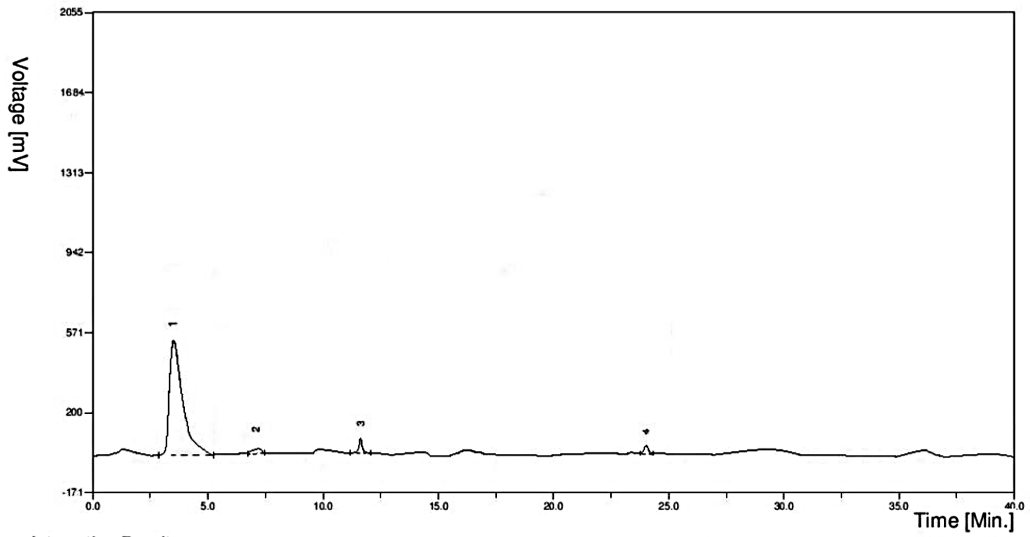

Integration Result

#	Name	Ret. Time(min)	Peak Height(mv)	Area(mv.sec)	Area Percentage(%)	Content(%)
1	Unknown	4.15	566.09	3999.47	83.520	0.0000
2	Unknown	7.06	9.78	118.09	2.402	0.0000
3	Unknown	12.02	18.47	377.57	7.680	0.0000
4	Unknown	24.11	7.67	314.53	6.398	0.0000
Total			602.01	4809.66	100	

HPLC chromatogram for hydrolysis of codrug **1** after 18 h

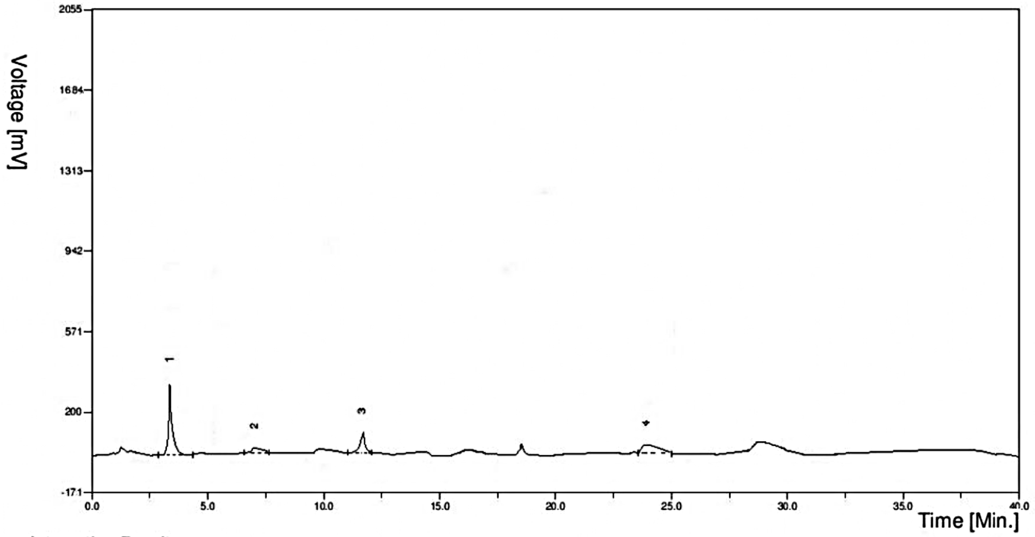

Integration Result

#	Name	Ret. Time(min)	Peak Height(mv)	Area(mv.sec)	Area Percentage(%)	Content(%)
1	Unknown	4.12	376.06	2633.92	56.220	0.0000
2	Unknown	7.04	27.29	326.64	6.972	0.0000
3	Unknown	12.03	43.98	899.61	19.202	0.0000
4	Unknown	24.14	19.75	824.84	17.606	0.0000
Total			467.08	4685.01	100	

HPLC chromatogram for hydrolysis of codrug **1** after 24 h

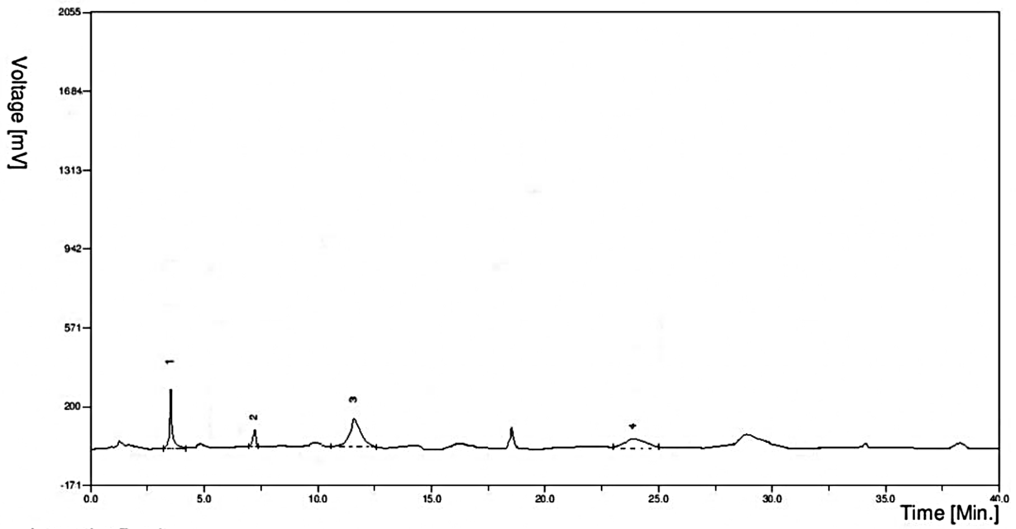

Integration Result

#	Name	Ret. Time(min)	Peak Height(mv)	Area(mv.sec)	Area Percentage(%)	Content(%)
1	Unknown	4.10	301.24	2099.66	30.660	0.0000
2	Unknown	7.05	67.33	326.64	11.991	0.0000
3	Unknown	12.05	108.18	2216.21	32.362	0.0000
4	Unknown	24.12	19.75	1711.09	24.986	0.0000
Total			496.50	6353.60	100	

HPLC chromatogram for hydrolysis of codrug **1** after 30 h

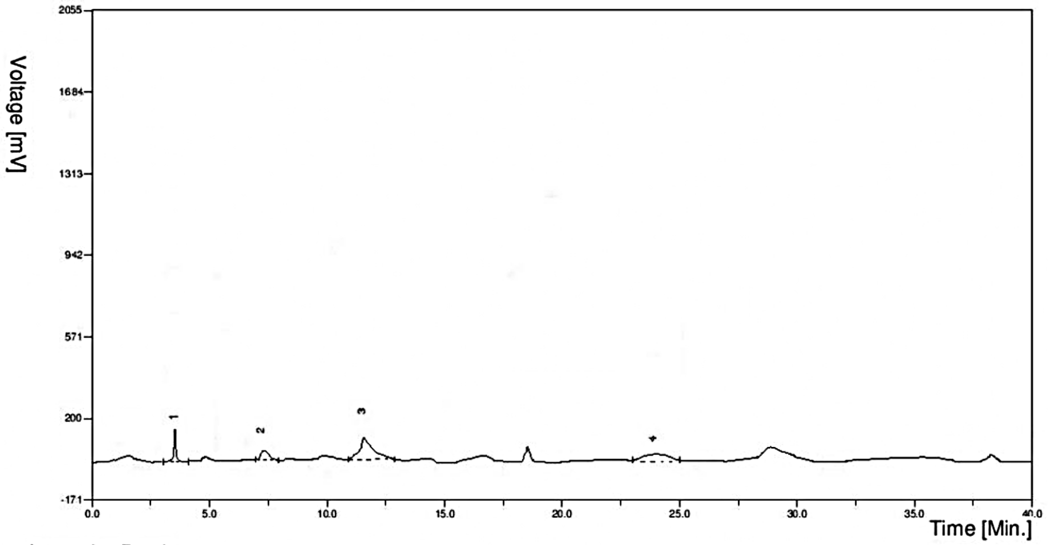

Integration Result

#	Name	Ret. Time(min)	Peak Height(mv)	Area(mv.sec)	Area Percentage(%)	Content(%)
1	Unknown	4.11	167.73	1171.99	16.882	0.0000
2	Unknown	7.06	90.59	1087.29	15.662	0.0000
3	Unknown	12.06	133.30	2733.09	39.369	0.0000
4	Unknown	24.11	48.89	2004.22	28.087	0.0000
Total			440.51	6996.59	100	

HPLC chromatogram for hydrolysis of codrug **1** after 36 h

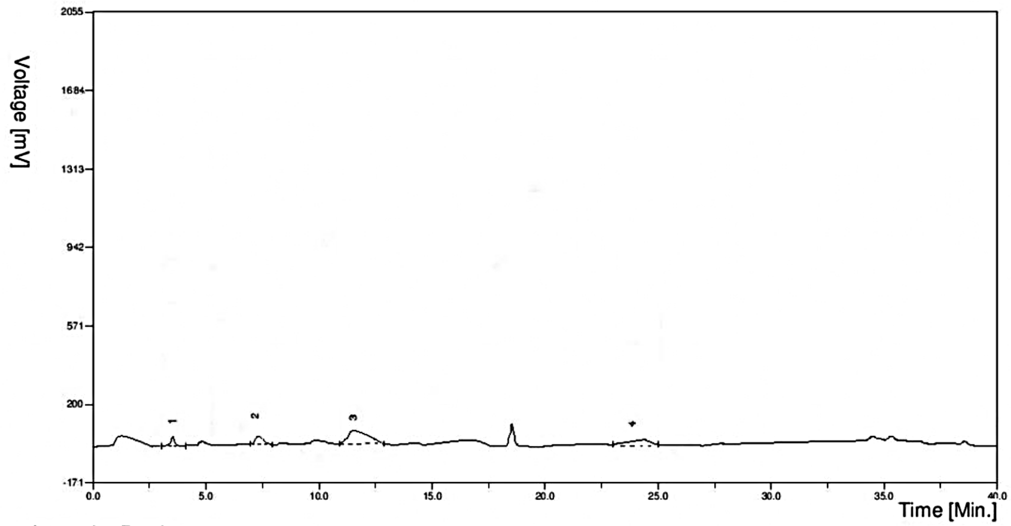

Integration Result

#	Name	Ret. Time(min)	Peak Height(mv)	Area(mv.sec)	Area Percentage(%)	Content(%)
1	Unknown	4.13	57.13	401.10	8.802	0.0000
2	Unknown	7.05	66.96	802.56	17.612	0.0000
3	Unknown	12.05	96.23	1971.41	43.262	0.0000
4	Unknown	24.12	33.70	1381.84	30.324	0.0000
Total			254.02	4556.91	100	

HPLC chromatogram for hydrolysis of codrug **1** after 42 h

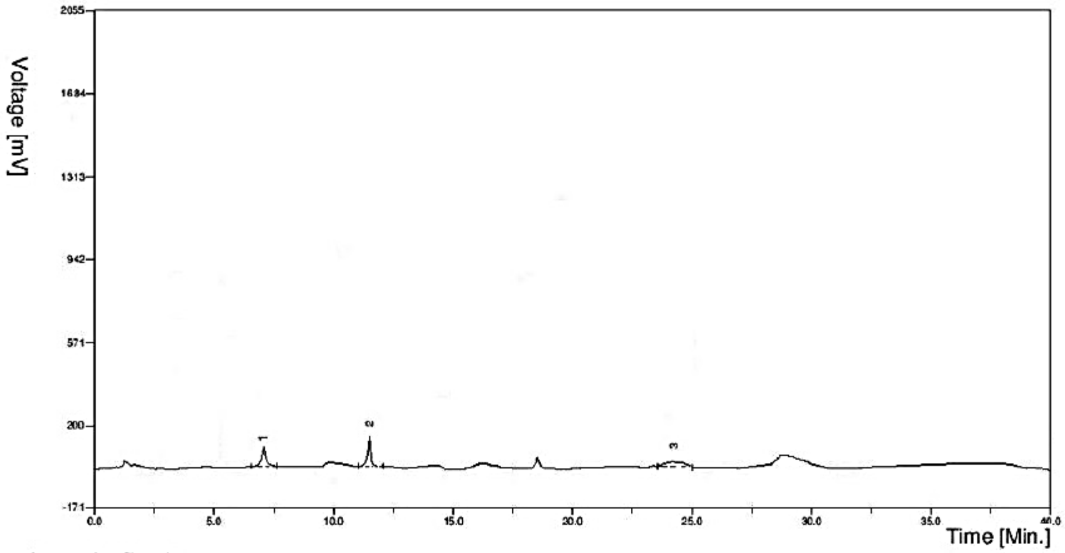

Integration Result

#	Name	Ret. Time(min)	Peak Height(mv)	Area(mv.sec)	Area Percentage(%)	Content(%)
1	Unknown	7.07	130.25	912.29	20.020	0.0000
2	Unknown	12.04	182.26	2190.59	48.072	0.0000
3	Unknown	24.08	35.57	1454.01	31.908	0.0000
Total			348.08	4556.89	100	

HPLC chromatogram for hydrolysis of codrug **1** after 48 h

Table S1. Area percentage from HPLC chromatograms for the sequential release of Pacitaxel (**4**) and Lenalidomide (**2**) from Codrug **1** over a time period of 0–48 h.

Entry	Time (h) measured	Codrug (R_f = ~ 4.0 min)	D-ribose (R_f = ~ 7.0 min)	Lenalidomide (R_f = ~ 12.0 min)	Paclitaxel (R_f = ~ 24.0 min)
1	0.0	100.0	0.0	0.0	0.0
2	6.0	96.28	0.200	0.960	2.560
3	12	93.12	0.802	2.880	3.198
4	18	83.52	2.402	7.680	6.398
5	24	56.22	6.972	19.20	17.60
6	30	30.66	11.99	32.36	24.98
7	36	16.88	15.66	39.36	28.08
8	42	8.802	17.61	43.26	30.32
9	48	0.0	20.02	48.07	31.90
